# Risk‐Based Prioritization of Organic Chemicals and Locations of Ecological Concern in Sediment From Great Lakes Tributaries

**DOI:** 10.1002/etc.5286

**Published:** 2022-02-28

**Authors:** Austin K. Baldwin, Steven R. Corsi, Owen M. Stefaniak, Luke C. Loken, Daniel L. Villeneuve, Gerald T. Ankley, Brett R. Blackwell, Peter L. Lenaker, Michelle A. Nott, Marc A. Mills

**Affiliations:** ^1^ US Geological Survey Boise Idaho; ^2^ US Geological Survey Middleton Wisconsin; ^3^ US Environmental Protection Agency Duluth Minnesota; ^4^ US Environmental Protection Agency Cincinnati Ohio

**Keywords:** Sediment toxicity, ToxCast, Organic chemicals, Mixed contaminants, Greak Lakes tributaries, Polycyclic aromatic hydrocarbons

## Abstract

With improved analytical techniques, environmental monitoring studies are increasingly able to report the occurrence of tens or hundreds of chemicals per site, making it difficult to identify the most relevant chemicals from a biological standpoint. For the present study, organic chemical occurrence was examined, individually and as mixtures, in the context of potential biological effects. Sediment was collected at 71 Great Lakes (USA/Canada) tributary sites and analyzed for 87 chemicals. Multiple risk‐based lines of evidence were used to prioritize chemicals and locations, including comparing sediment concentrations and estimated porewater concentrations with established whole‐organism benchmarks (i.e., sediment and water quality criteria and screening values) and with high‐throughput toxicity screening data from the US Environmental Protection Agency's ToxCast database, estimating additive effects of chemical mixtures on common ToxCast endpoints, and estimating toxic equivalencies for mixtures of alkylphenols and polycyclic aromatic hydrocarbons (PAHs). This multiple‐lines‐of‐evidence approach enabled the screening of more chemicals, mitigated the uncertainties of individual approaches, and strengthened common conclusions. Collectively, at least one benchmark/screening value was exceeded for 54 of the 87 chemicals, with exceedances observed at all 71 of the monitoring sites. Chemicals with the greatest potential for biological effects, both individually and as mixture components, were bisphenol A, 4‐nonylphenol, indole, carbazole, and several PAHs. Potential adverse outcomes based on ToxCast gene targets and putative adverse outcome pathways relevant to individual chemicals and chemical mixtures included tumors, skewed sex ratios, reproductive dysfunction, hepatic steatosis, and early mortality, among others. The results provide a screening‐level prioritization of chemicals with the greatest potential for adverse biological effects and an indication of sites where they are most likely to occur. *Environ Toxicol Chem* 2022;41:1016–1041. Published 2022. This article is a U.S. Government work and is in the public domain in the USA. *Environmental Toxicology and Chemistry* published by Wiley Periodicals LLC on behalf of SETAC.

## Graphical abstract

Relative priority for bioeffects due to organic chemical exposure in sediment from Great Lakes tributaries.

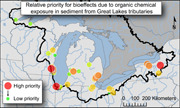

## INTRODUCTION

Organic chemicals are used in many industrial, agricultural, and household applications. These chemicals (and associated metabolites/degradates) include herbicides, pharmaceuticals, flame retardants, flavors and fragrances, detergent metabolites, fuels, polycyclic aromatic hydrocarbons (PAHs), and others. They can enter waterways through atmospheric deposition, stormwater runoff, wastewater treatment plant discharge, combined sewer overflows, agricultural runoff, leaching landfills, septic systems, and sanitary sewer infrastructure (Barber et al., 2015; Kolpin et al., 2002). Many organic chemicals bind to organic particles and accumulate in sediment (Koelmans et al., 2006). Subsequent chemical partitioning between sediment particulates and porewater can result in desorption of these chemicals into the water column through bioturbation or diffusion (Alvarez et al., 2012; Remaili et al., 2017). These processes make the sediment bed important as a secondary source of chemical contamination to the aquatic environment, particularly for certain legacy contaminants that have had decreasing environmental inputs following stricter control and legislative action (Venier et al., 2014). Many organic chemicals can cause toxicity through different biological pathways, posing a potential threat to aquatic life (Barber et al., 2015; Vajda et al., 2008), sometimes at environmentally relevant concentrations.

Monitoring efforts have traditionally focused on reporting the occurrence and concentration of contaminants in the environment (Elliott et al., 2017; Tertuliani et al., 2008; Venier et al., 2014). Advances in high‐resolution analytical instrumentation have provided the ability to measure an increasing number of chemicals at ng/L concentrations in water. Although this type of monitoring is important in detecting contaminants, it provides no context for potential biological effects, so the ecological implications of chemicals measured in aquatic systems often remain poorly understood (Blackwell et al., 2017; Bradley, Romanok, et al., 2020; Judson et al., 2009). Because organic chemicals rarely occur in isolation (Bradley et al., 2019; Elliott et al., 2017) and some may interact, leading to unexpected adverse outcomes (Marinovich et al., 1996; Thrupp et al., 2018), the presence of chemical mixtures can complicate attempts to understand risk (Schoenfuss et al., 2016). Thus, there is a need to clarify what concentrations and mixtures of chemicals may be hazardous from a biological perspective.

The goal of the present study was to identify locations and chemicals of concern in Great Lakes (USA/Canada) tributaries by examining the occurrence and potential biological effects of 87 organic chemicals in bed sediment. A risk‐based, multiple‐lines‐of‐evidence approach was used to prioritize chemicals and locations to maximize the number of chemicals screened, mitigate uncertainties of individual methods, and strengthen common conclusions. We considered the likelihood for these chemicals to partition into the sediment porewater, as well as additional factors including predicted porewater concentrations, relative toxicological or pathway‐specific chemical potency, association of potentially impacted pathways with adverse outcomes of ecological significance, and watershed attributes. During 2017, sediment samples were collected at 71 Great Lakes tributary locations spanning a gradient of watershed land cover types. Chemical concentrations were compared with whole‐organism sediment quality benchmarks and screening values. Porewater concentrations estimated from sediment concentrations were compared with whole‐organism water quality benchmarks and screening values, as well as with in vitro screening values from the ToxCast database (US Environmental Protection Agency [USEPA], 2017) to assess the potential for bioeffects. The results from our study provide a baseline for future monitoring and highlight chemicals and locations for additional studies.

## MATERIALS AND METHODS

### Site selection

**Figure 1:**
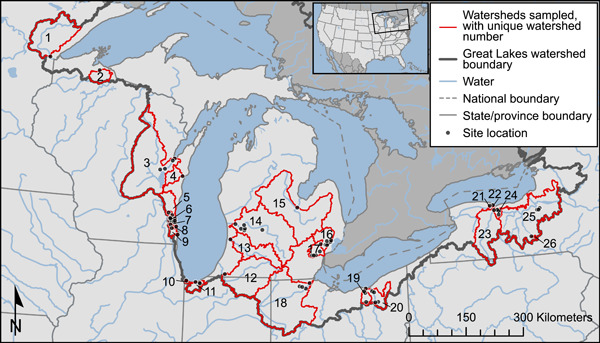
Map of the Great Lakes Basin and the sampled watersheds, modified from Baldwin et al. (2020). Watershed numbers correspond with map numbers in Table 1. Base map is compiled from North American hydrology and political boundaries (Instituto Nacional de Estadística Geografía e Informática et al., 2006a, 2006b), as well as the Great Lakes basin boundary (Grannemann, 2010). Site watershed boundaries were determined using linework from the Watershed Boundary Dataset and catchments from the medium‐resolution NHDPlus V2 Dataset (US Department of Agriculture—Natural Resources Conservation Service et al., 2009; USEPA and U.S Geological Survey, 2012).

Samples of streambed sediment were collected from Great Lakes tributaries in Minnesota, Wisconsin, Indiana, Michigan, Ohio, and New York in June–July 2017. One to seven locations within 26 watersheds were sampled, for a total of 71 sampling locations (Table 1 and Figure 1, and Supporting Information, Table S1). Locations were chosen to represent a broad range of watershed drainage areas (3.5–16 300 km^2^), land uses (0.7%–100% urban; 0%–90% agricultural; 0%–84% undeveloped/natural), population densities (2.8–2260 people/km^2^), percentage impervious (0.2%–72%), and wastewater contributions (0%–48% of streamflow).

**Table 1:** Sampling locations and basin statistics for Great Lakes tributaries sampled for organic chemicals in sediment, 2017^a^

Lake	Watershed (map no.)	Site name	Site abbreviation	Drainage area (km^2^)	Population density (people/km^2^)	% Impervious
Superior	Saint Louis (1)	Saint Louis River at Scanlon, MN	MN‐SLR	8890	9.2	0.5
	Bad (2)	Bad River near Odanah, WI	WI‐BRO	1545	2.8	0.2
Michigan	Fox (3)	Garners Creek at Park St. at Kaukauna, WI	WI‐GCK	21	834	30
		East River below Cedar St. at Green Bay, WI	WI‐ERG	381	200	7.1
		West Branch Mud Creek below CTH BB at Appleton, WI	WI‐WMC	26	175	17
		Ashwaubenon Creek above Parkview Rd. at De Pere, WI	WI‐ACA	75	106	10
	Manitowoc (4)	Manitowoc River at Manitowoc, WI	WI‐MAM	1343	25	1.6
	Milwaukee (5)	Milwaukee River at Milwaukee, WI	WI‐MIE	1785	195	6.0
		Milwaukee River at Mouth at Milwaukee, WI	WI‐MIM	2240	434	12
		Milwaukee River at Walnut St. at Milwaukee, WI	WI‐MIP	1804	233	6.5
		Northridge Lake near Milwaukee, WI	WI‐NRL	3.5	1441	49
	Menomonee (6)	Menomonee River at CTH F near Germantown, WI	WI‐MEF	29	67	2.3
		Menomonee River at Butler, WI	WI‐MEB	154	387	18
		Little Menomonee River at Lovers Ln. at Milwaukee, WI	WI‐LML	55	634	19
		Menomonee River above Church St. at Wauwatosa, WI	WI‐MEC	288	579	23
		Menomonee River near N. 25th St. at Milwaukee, WI	WI‐MET	355	966	28
		Menomonee River at Ridge Blvd. at Wauwatosa, WI	WI‐MER	233	525	21
		Underwood Creek at Juneau Blvd. at Elm Grove, WI	WI‐UCJ	23	520	21
	Kinnickinnic (7)	Kinnickinnic River at Lincoln Ave. at Milwaukee, WI	WI‐KKL	62	2265	51
	Oak (8)	Oak Creek at Mill Pond at South Milwaukee, WI	WI‐OCM	69	739	31
	Root (9)	Root River at Layton Ave. at Greenfield, WI	WI‐RRL	31	1150	32
		Root River near Franklin, WI	WI‐RRR	127	830	25
		Root River near Clayton Park at Racine, WI	WI‐RRC	506	334	12
	Indiana Harbor Canal (10)	Indiana Harbor Canal at East Chicago, IN	IN‐IHC	100	914	47
	Burns Ditch (11)	Portage‐Burns Waterway at Portage, IN	IN‐PBW	857	345	14
		Coffee Creek DS of 1100 N. near Chesterton, IN	IN‐CCU	32	68	3.4
		Coffee Creek at Chesterton, IN	IN‐CCD	40	122	6.4
	St. Joseph (12)	St. Joseph River at Niles, MI	MI‐SJO	9628	80	3.8
	Kalamazoo (13)	Kalamazoo River at New Richmond, MI	MI‐KAL	5122	91	3.5
	Grand (14)	Peacock Ditch at Grand River Ave. near Ionia, MI	MI‐PEA	15	9.0	1.5
		Indian Mill Creek at Turner Ave. at Grand Rapids, MI	MI‐IND	44	297	16
		Plaster Creek at 28th St. at Grand Rapids, MI	MI‐PLS	119	468	27
		Tributary to Buck Creek at Division Ave. at Wyoming, MI	MI‐TBC	16	1396	48
		Buck Creek at State Hwy. M‐21 at Grandville, MI	MI‐BCK	131	761	30
		Grand River at Eastmanville, MI	MI‐GRE	13 560	109	4.3
Huron	Saginaw (15)	Saginaw River at Saginaw, MI	MI‐SAG	15 509	69	3.0
Erie	Clinton (16)	Clinton River at Sterling Heights, MI	MI‐CLT	803	443	16
		Red Run at Ryan Rd. near Warren, MI	MI‐RRR	89	1734	52
		Bear Creek immediately DS at Miller Drain at Warren, MI	MI‐BAR	48	1518	72
		Red Run at 15 Mile Rd. at Sterling Heights, MI	MI‐RRS	275	1609	53
		North Branch Clinton River near Mt. Clemens, MI	MI‐NBC	512	84	3.7
		Clinton River at Moravian Dr. at Mount Clemens, MI	MI‐CRM	1937	611	21
	Rouge (17)	River Rouge at Birmingham, MI	MI‐RRB	95	658	24
		River Rouge at Detroit, MI	MI‐RRD	476	965	34
		Lower River Rouge at Beck Rd. near Sheldon, MI	MI‐LRB	24	242	7.9
		Lower River Rouge at Haggerty Rd. at Wayne, MI	MI‐LRH	95	376	16
		Lower River Rouge at Wayne Road at Wayne, MI	MI‐LRW	183	595	23
	Maumee (18)	Maumee River at Waterville, OH	OH‐MRW	16 295	54	2.4
		Swan Creek at Toledo, OH	OH‐SCT	519	174	6.9
		Swan Creek at Oak Openings Metropark, OH	OH‐SCO	232	57	2.3
		Swan Creek at Township Road EF near Swanton, OH	OH‐SCE	65	49	2.0
	Rocky (19)	West Branch Rocky River near Medina, OH	OH‐WBR	158	323	10
		Rocky River near Berea, OH	OH‐RRB	692	358	9.5
		Rocky River above STP near Lakewood, OH	OH‐RRS	755	408	11
		East Branch Rocky River at W. Center St., Berea, OH	OH‐EBR	193	441	10
	Cuyahoga (20)	Cuyahoga River at Old Portage, OH	OH‐CRP	1047	297	9.3
		Cuyahoga River at Independence, OH	OH‐CRI	1836	326	11
		West Creek at Independence, OH	OH‐WCI	35	1130	28
		Cuyahoga River at Munroe Falls, OH	OH‐CRM	841	159	5.1
		Tinkers Creek at Dunham Rd. near Independence, OH	OH‐TCD	246	462	20
Ontario	Northrup (21)	Northrup Creek at North Greece, NY	NY‐NCG	26	294	5.6
	Slater (22)	Slater Creek at Hojack Industrial Park at Mount Read, NY	NY‐SCH	12	1610	25
	Genesee (23)	Genesee River at Ford St. Bridge at Rochester, NY	NY‐GRF	6403	45	1.2
	Irondequoit (24)	Irondequoit Creek at Railroad Mills near Fishers, NY	NY‐ICR	100	78	2.4
		Allen Creek near Rochester, NY	NY‐ACR	80	758	18
		Irondequoit Creek above Blossom Rd. near Rochester, NY	NY‐ICB	364	442	8.9
		Thomas Creek at East Rochester, NY	NY‐TCR	74	367	5.4
	Oswego (25)	Harbor Brook at Hiawatha Blvd., Syracuse, NY	NY‐HBK	31	782	16
		Geddes Brook at Fairmount, NY	NY‐GBF	22	594	15
		Ley Creek at Lemoyne and Factory at Mattydale, NY	NY‐LEY	62	812	34
	Cascadilla (26)	Cascadilla Creek at Ithaca, NY	NY‐CCI	37	150	2.3

^a^Watershed map numbers refer to watershed numbers in Figure 1. Watershed population density calculated from 2010 census block data (US Census Bureau Geography Division, 2010); mean percentage impervious surfaces calculated from 2011 National Land Cover Dataset (Homer et al., 2015); drainage area calculated from the 2012 conterminous wall‐to‐wall anthropogenic land use trends (NWALT) dataset (Falcone, 2015); methods for calculating all watershed statistics are described elsewhere (Baldwin et al., 2020).

MI = Michigan; OH = Ohio; IN = Indiana; WI = Wisconsin; NY = New York; MN = Minnesota; DS = downstream; STP = sewage treatment plant; CTH = county trunk highway; km^2^ = square kilometers.

### Sample collection and analysis

Sample collection methods were described previously (Baldwin et al., 2020) and are summarized in this paragraph. Sediment samples were collected either by boat or while wading in the stream, targeting depositional areas with fine‐grained sediments (silts). A push core sampler (WaterMark® Universal Core Head Sediment Sampler; Forestry Suppliers) with a polycarbonate tubing (Forestry Suppliers; 70‐mm outer diameter × 66.7‐mm inner diameter) was used to collect sediment from the surface to a depth of 15 cm. The 15‐cm depth was used to focus on recently deposited sediments. The sediment core was placed into a stainless‐steel pan, divided vertically, and the halves were transferred to separate baked amber‐glass jars. Samples were stored in the dark on ice, and within 48 h were shipped for chemical analyses. A new core tube was used at each sampling location. Between sampling locations, sediment processing equipment was cleaned using detergent (Alconox®) water followed by three rinses with tap water and three rinses with deionized water.

One‐half of each sediment core was analyzed for 51 organic chemicals (Supporting Information, Table S2) representing 13 chemical classes (e.g., detergent metabolites, flavors and fragrances, fire retardants, solvents, herbicides, insecticides; described in the *Data analysis* section) by the US Geological Survey (USGS) National Water Quality Laboratory using a pressurized solvent‐extraction system followed by capillary‐column gas chromatography–mass spectrometry (GC–MS; Burkhardt et al., 2006). The target chemicals were considered to be good indicators of industrial or domestic wastewater, and/or chemicals of human or environmental health concern (Kolpin et al., 2002; Zaugg et al., 2006). Laboratory recoveries of chemicals analyzed over the course of the present study period are summarized in the Supporting Information, Table S3. Mean recoveries were in the 70%–130% range for 79% of chemicals, and in the 20%–69% range for 19% of chemicals. Sample results were not adjusted for recovery rates.

The other half of each core was analyzed by Battelle Memorial Institute (Stony Brook, NY, USA) for 36 organic chemicals in an additional (14th) chemical class: PAHs (18 parent and 18 alkylated PAHs; Supporting Information, Table S2). The PAHs were determined via GC–MS in selected ion monitoring mode, described in detail in Baldwin et al. (2020). The PAH results were published previously (Baldwin et al., 2020) but are included in the present study for assessment of potential biological effects as part of the full chemical mixture. A sample split was used for analysis (by ALS Environmental, Kelso, WA, USA) of total organic carbon (TOC) using a CM5012 CO_2_ Coulometer (UIC) and a modified ASTM International (2020) method D4129‐05. Laboratory reporting levels for organic chemicals are summarized in the Supporting Information, Table S2.

Field duplicate samples were collected as separate samples (rather than splits) at six locations for organic chemicals not including PAHs, and at eight locations for PAHs. Relative percent differences (RPDs) between duplicates varied by chemical, with median RPDs ranging from 2.2% to 59.4% (Supporting Information, Table S4).

### Data analysis

Laboratory reporting limits for each chemical are summarized in the Supporting Information, Table S2. Concentrations less than the reporting limit were substituted with zero in summations of total sample concentrations and total chemical class concentrations. The 87 organic chemicals were aggregated into 14 chemical classes: antimicrobial disinfectants (*n* = 5), antioxidants (1), detergent metabolites (i.e., alkylphenols/surfactants) (8), dyes and pigments (1), fire retardants (4), flavors and fragrances (10), fuels (4), herbicides (4), insecticides (4), nonprescription drugs (1), PAHs (36), plasticizers (4), solvents (1), and sterols (4) (Supporting Information, Table S2). The classes were modified from aggregations used in previous studies (Baldwin et al., 2016; Corsi et al., 2019; Sullivan et al., 2005). These class assignments are imperfect because some chemicals have numerous uses and could fit into multiple classes. One notable example is carbazole: although classified in the present study as an insecticide, carbazole has a wide range of uses and potential sources, some of which may be more important than its use in insecticides (Arbiser et al., 2006; Zaugg et al., 2006). Even so, the use of these chemical classes aids in interpretation of results and comparison across studies.

#### Estimation of porewater concentrations

Porewater concentrations were estimated in the following equation for comparison with water quality benchmarks and screening values, and with activity concentrations at cutoff (ACCs) reported for ToxCast high‐throughput screening data.

(1) $$C_{\mathrm{PW}}=C_{\mathrm{SED}}/\left(K_{\mathrm{OC}}\times f_{\mathrm{OC}}\right)$$

where *C*_PW_ is the dissolved porewater concentration (µg/L); *C*_SED_ is the total sediment concentration (µg/kg, dry wt); *K*_OC_ is the chemical‐specific organic carbon–water partition coefficient (L/kg); and *f*_OC_ is the mass fraction of organic carbon in the sediment sample.

The *C*_SED_ and *f*_OC_ values were measured directly. The chemical‐specific *K*_OC_ values, which relate to the sorption properties between chemicals and organic matter in soil or sediment, were obtained from the literature (Hawthorne et al., 2007; Mansouri et al., 2018; Williams et al., 2017; USEPA, 2021a; Supporting Information, Table S2). Literature *K*_OC_ values were not found for four chemicals (C1‐naphthalene, C2‐fluoranthene/pyrene, C3‐fluoranthene/pyrene, and C4‐benz[*a*]anthracene/chrysene), precluding estimation of their porewater concentrations.

#### Assessment of potential bioeffects of individual chemicals

Three different approaches were used to assess the potential for adverse biological effects of individual chemicals: (1) sediment toxicity quotients (TQs), (2) porewater TQs, and (3) porewater exposure–activity ratios (EARs; Table 2). The use of multiple approaches maximized the number of chemicals included in the overall assessment and strengthened the combined conclusions over those from individual approaches.

**Table 2:** Definitions of toxicity quotient and exposure–activity ratio summations used for assessment of potential biological effects

Summation	Abbreviation	Description
Toxicity quotient	TQ	The ratio of the measured concentration of a chemical in a sample and the sediment or water quality benchmark for that chemical
Maximum TQ	*TQ*_Max_	The maximum TQ for a given chemical in a sample
TQ by chemical class	*TQ*_Class_	The sum of the *TQ*_Max_ values for all chemicals in a sample in a common chemical class
TQ by sample	*TQ*_Sample_	The sum of the *TQ*_Max_ values for all chemicals in a sample
Exposure–activity ratio	EAR	The ratio of the estimated porewater chemical concentration and the ToxCast activity concentration at cutoff
Maximum EAR	*EAR*_Max_	The maximum EAR for a given chemical in a sample
EAR by chemical class	*EAR*_Class_	The sum of the *EAR*_Max_ values for all chemicals in a sample in a common chemical class
EAR by sample	*EAR*_Sample_	The sum of the *EAR*_Max_ values for all chemicals in a sample
EAR by assay endpoint	*EAR*_Endpoint_	The sum of the EAR values for all chemicals associated with a common ToxCast assay endpoint

#### Sediment and porewater TQs

Comparison of environmental contaminant concentrations with benchmark values is a common method of assessing the potential for biological impacts (Diamond et al., 2011; Hull et al., 2015). Established whole‐organism sediment and water quality benchmarks and screening values for individual chemicals (collectively termed benchmarks hereafter) were compiled from US and Canadian government agencies and the literature (Supporting Information, Tables S5 and S6). In many cases multiple benchmarks were found for a single chemical, sometimes spanning up to 1 or more orders of magnitude and thus representing varying degrees of impact/protection. For each chemical at each site, TQs were calculated as the ratio of the measured chemical concentration and each available sediment quality benchmark. The TQ representing the most sensitive (i.e., lowest) sediment quality benchmark for a given chemical at a site was identified as the *TQ*_Max_ (Baldwin et al., 2016; Corsi et al., 2019; Diamond et al., 2011; Table 2). Porewater TQ and *TQ*_Max_ values were calculated under the same basic approach but using estimated porewater concentrations and water quality benchmarks. A TQ greater than 1.0 means the concentration exceeds the benchmark, indicating the potential for adverse biological effects. As with the other assessment methods used in our study, TQ values are intended only as a screening tool. Furthermore, a TQ less than 1.0 does not eliminate the possibility of adverse biological effects because, for example, multiple chemicals occurring at low concentrations may act together in an additive manner (Marinovich et al., 1996; Thrupp et al., 2018).

To be protective of the most sensitive species, *TQ*_Max_ values were used for analyses in the present study. Sediment and porewater *TQ*_Max_ values were summed for the chemicals in each chemical class within a sample to obtain sediment *TQ*_Class_ and porewater *TQ*_Class_, and for all the chemicals in the sample to obtain sediment *TQ*_Sample_ and porewater *TQ*_Sample_. These summations were meant for screening purposes only, because the toxicity of multiple chemicals is not necessarily additive, and benchmarks are not always derived from consistent in vivo tests.

#### EARs

Estimated porewater concentrations were also compared with values from the USEPA (2017) ToxCast database Ver 3.2, which contains chemical screening data from in vitro high‐throughput assays capturing dozens of biological pathways, for thousands of chemicals. The screening assays incorporated in ToxCast capture a range of biological activities that, in some cases, can result in chronic and sublethal impacts like endocrine disruption, impacts on energy metabolism pathways, and various cellular stress responses that may not be detected in conventional aquatic toxicity testing, but may nonetheless have implications for ecological fitness. Although not all biological activities measured in ToxCast will necessarily be adverse, the broad pathway coverage can help define a lower bound potency estimate (Paul Friedman et al., 2020) that may be useful in a screening context, particularly when other data are lacking. A thorough description of the ToxCast data analysis pipeline may be found in Filer et al. (2016).

ToxCast provides several summary metrics derived from dose–response curves to indicate chemical potency. Following previous studies (Alvarez et al., 2021; Blackwell et al., 2017; Bradley et al., 2019; Corsi et al., 2019), the present study used the ACC as an indicator of the chemical concentration that may be required for bioactivity to begin occurring in the assay. The ACC is an assay‐specific metric determined as a multiplier of the baseline median absolute deviation of measured activity in the assay that provides an indication of the concentration at which the bioactivity measured first exceeds the baseline concentration. The ACC value was used as the endpoint to compare estimated porewater concentrations for all available chemical assays. The EARs for individual chemicals were calculated by dividing the estimated porewater chemical concentration by the ACC for each assay (Table 2). The EARs were computed using the R package *toxEval* (DeCicco et al., 2018), which was designed to prioritize chemicals of concern and develop a better understanding of the potential biological relevance of environmental chemistry data. The Supporting Information includes a discussion of ToxCast results that were omitted from the analysis, as well as the ToxEval input file with estimated porewater concentrations for each chemical at each site (Supporting Information, Table S9).

The maximum EAR value for a given chemical in a sample (*EAR*_Max_) was used for most analyses to be conservative and minimize false negatives. An *EAR*_Max_ value of 0.001 was used as a threshold for identifying chemicals with potential effects. This value has been used by previous studies (Bradley, Romanok, et al., 2020; Bradley, Journey, et al., 2020) and has been shown to be a level of potential concern based on comparison with established water quality benchmarks (Corsi et al., 2019). The *EAR*_Max_ values were summed for the chemicals in each chemical class to get *EAR*_Class_, and for all the chemicals in the sample to get *EAR*_Sample_ (Blackwell et al., 2017). These summations across chemicals were not specific to a common ToxCast endpoint, but generally represented multiple different endpoints for use as an initial screening value. (Summations of *EAR*_MAX_ for common endpoints were also done and are described in the *EAR mixtures* section). As with the *TQ*_Class_ and *TQ*_Sample_ values just described in the *Sediment and porewater TQs* section, these summations were meant for screening purposes only and would not be expected to accurately represent the bioeffect potential of mixtures. In general, EARs are best viewed as a relative ranking tool that considers differences in chemical concentrations and endpoint‐specific potencies.

#### Availability of established benchmarks and ToxCast ACC values

**Figure 2:**
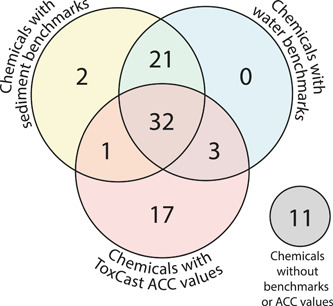
The number of organic chemicals analyzed in sediment samples from Great Lakes tributaries in 2017 with established whole‐organism sediment quality and porewater quality benchmarks and in vitro ToxCast activity concentration at cutoff (ACC) values.

The use of three different approaches to assess the potential for adverse biological effects from individual chemicals enabled us to screen 76 of the 87 chemicals detected with at least one approach. Of the 87 chemicals analyzed, established sediment quality benchmarks were found for 56 chemicals. Water quality benchmarks were found for 56 chemicals as well, although not for all the same chemicals as the sediment quality benchmarks (Figure 2). The ToxCast database contained ACC values for 59 chemicals; however, 6 were excluded because of data quality flags or poor dose–response curves, leaving 53 chemicals for which EARs were calculated. The ToxCast database included ACC values for 17 chemicals that otherwise lacked sediment or water quality benchmarks. We were able to screen 32 chemicals using all three approaches, and 11 chemicals had no benchmarks or ACCs available.

#### Potential biological effects of chemical mixtures

Chemical‐specific TQs and EARs are useful for identifying chemicals of interest, but likely underestimate potential biological effects at a given location, because chemicals commonly occur as complex mixtures (Bradley et al., 2019; Elliott et al., 2018), which could cause, for example, additive effects (Marinovich et al., 1996; Thrupp et al., 2018). In the previous sections several additive methods for estimating the potential biological effects of chemical mixtures in individual samples were described (i.e., *TQ*_Class_, *EAR*_Class_, *TQ*_Sample_, *EAR*_Sample_). Those approaches are useful for screening purposes because they account for the potential toxicity from a wide variety of chemicals, but their assumed additivity may overestimate biological effects in cases of chemical mixtures with multiple modes of action (Faust et al., 2003). The following section describes several approaches that use a common benchmark and mode of action to assess the potential for adverse biological effects from mixtures of chemicals. Although these approaches are relatively narrow in focus (limited to only a subset of chemicals present), the use of a common benchmark and mode of action provides greater confidence in the meaning of the results.

#### PAHs

The potential toxicity of PAH mixtures in these samples was assessed in Baldwin et al. (2020) and is included in the present study as part of a more comprehensive toxicity assessment. Mixtures of PAHs were assessed using two methods. The first method involved comparisons of sediment concentrations with the consensus‐based probable effect concentration (PEC) and threshold effect concentration (TEC; Ingersoll et al., 2001; Kemble et al., 2013; MacDonald et al., 2000). The PEC and TEC values are 22 800 and 1610 µg/kg, respectively, for the combined concentration of the 16 USEPA Priority Pollutant PAHs (∑*PAH*_16_; includes naphthalene, acenaphthalene, acenaphthene, fluorene, anthracene, phenanthrene, pyrene, fluoranthene, benz[*a*]anthracene, chrysene, benzo[*a*]pyrene, benzo[*b*]fluoranthene, benzo[*k*]fluoranthene, indeno[1,2,3‐*cd*]pyrene, benzo[*ghi*]perylene, and dibenz[*a,h*]anthracene). A PEC quotient (PECQ) and a TEC quotient (TECQ) were calculated for each sample by dividing the sediment ∑*PAH*_16_ concentration (not TOC‐normalized; Van Metre & Mahler, 2010) by the respective PEC or TEC. Adverse biological effects were considered likely for samples with PECQ greater than 1.0, unlikely for samples with TECQ less than 1.0, and possible for samples with TECQ greater than 1.0 but PECQ less than 1.0 (Ingersoll et al., 2001).

The second method to assess the potential toxicity of mixtures of PAHs was using the sum equilibrium partitioning sediment benchmark toxicity unit (∑ESBTU), which accounts for the varying bioavailability of individual PAH chemicals in different sediments (USEPA, 2003). The ∑ESBTU for each sample was calculated by dividing the TOC‐normalized concentrations of 35 PAHs (all the PAHs listed in the Supporting Information, Table S2, except for C2‐fluoranthene/pyrene) by chemical‐specific final chronic values and summing the results. Values of ∑ESBTU greater than 1.0 indicate the potential for adverse biological effects (i.e., narcosis) on sensitive benthic organisms (USEPA, 2003).

#### Alkylphenols

Many alkylphenols (chemicals in the class termed detergent metabolites in the present study) share a common mode of toxicity (narcosis; Schüürmann, 1991), and thus additivity of their effects is likely (Canadian Council of Ministers of the Environment [CCME], 2002). The potential biological effect of alkylphenol mixtures was assessed using a toxic equivalency (TEQ) approach, which sums the toxicities of individual chemicals relative to that of nonylphenol, using the following equation (CCME, 2002):

(2) $$\mathrm{TEQ}=\Sigma \left(C_{i}\times TEF_{i}\right)$$

where TEQ is the concentration of the mixture of alkylphenolic chemicals expressed as the toxic equivalent of nonylphenol; *C*_*i*_ is the concentration of chemical *i*; and *TEF*_*i*_ is the TEQ factor for chemical *i*.

The TEFs for the chemicals in the class detergent metabolites were from the CCME (2002), except for 4‐tert‐octylphenol and 4‐cumylphenol, for which a TEF of 0.5 was assumed based on the TEFs of other chemicals. (The TEFs were 0.5 for 4‐tert‐octylphenol monoethoxylate, 4‐tert‐octylphenol diethoxylate, 4‐nonylphenol monoethoxylate, and 4‐nonylphenol diethoxylate). An organic carbon‐adjusted alkylphenol TQ for each site was calculated using the following equation (CCME, 2002):

(3) AlkylphenolTQ=TEQ/(1400×TOC)

where 1400 is the freshwater interim sediment quality guideline, and TOC is the site‐specific total organic carbon, in percentage.

Alkylphenol TQ values greater than 1.0 indicate the potential for adverse biological effects (i.e., narcosis) on sensitive benthic organisms. The results are likely biased low, because concentrations below the reporting limit were assumed to equal zero. Importantly, this assessment is focused on narcosis and does not account for the potential endocrine activity of alkylphenols. (Endocrine activity is, however, included in the EAR‐based approach).

#### EAR mixtures

Potential effects of chemical mixtures were also assessed using estimated porewater concentrations and calculated EAR values. A benefit to the EAR approach is the ability to combine the effects of multiple chemicals on the thousands of in vitro responses in ToxCast. For each ToxCast assay endpoint, EAR values were summed within an assay across all chemicals to calculate *EAR*_Endpoint_, following previously defined approaches (Blackwell et al., 2017; Corsi et al., 2019). The *EAR*_Endpoint_ value assumes additivity and represents the combined effect of all detected chemicals in a sample on each in vitro response. To identify sites/samples where the cumulative mixture of chemicals present may be of greatest concern, sites/samples were ranked by *EAR*_Endpoint_, and an arbitrary cutoff of *EAR*_Endpoint_ greater than 0.1 was used in at least 20% of the sites to prioritize mixtures of concern. Chemicals that contributed at least 10% of the EAR benchmark threshold of 0.01 were considered part of the mixture for each endpoint.

Most ToxCast assays do not provide a direct measure of impacts on survival, growth, and reproduction, the endpoints typically considered in ecological risk assessment. Consequently, more detailed understanding of the biological relevance of the specific proteins, biochemical reactions, pathways, and so on, is required to interpret the potential significance to ecological hazards. To accomplish this, the gene target annotations associated with ToxCast assays that were prevalent in the present study were used to infer potentially affected biological implications in two ways. First, some gene targets have been linked to adverse outcome pathways (AOPs; Society for the Advancement of Adverse Outcome Pathways [SAAOP], 2018). The AOP framework assembles biological understanding and evidence linking perturbation of specific molecular targets to adverse outcomes of ecological significance (Ankley et al., 2010). Thus, ToxCast assays associated with specific AOPs (Fay et al., 2018; Mortensen et al., 2018, 2021; Pittman et al., 2018; SAAOP, 2018) have previously been used in assessment of water quality data to help interpret the significance of EAR results for estimation of potential ecological impacts (Ankley et al., 2021; Corsi et al., 2019). The ToxCast assays associated with priority chemicals and priority chemical mixtures for the present study were then mapped to associated AOPs (Supporting Information, Table S10) to identify potential adverse outcomes relevant to the monitored chemicals.

Second, gene ontology information for ToxCast assay targets with relevance for priority chemicals/chemical mixtures was mined from the Database for Annotation, Visualization and Integrated Discovery (DAVID; Huang et al., 2009; Laboratory for Human Retrovirology and Immunoinformatics, 2020) and the Protein Analysis Through Evolutionary Relationships (PANTHER) classification system (Huaiyu et al., 2020; PANTHER 2021). The primary aim, for the present study, was to draw inferences as to potential apical effects in organisms exposed to the measured chemicals. Because most ToxCast assays target human‐relevant endpoints, we considered gene orthologs of the nonmammalian vertebrate species *Danio rerio* (zebrafish) and *Xenopus tropicalis* (western clawed frog)—two aquatic model organisms—in addition to *Homo sapiens*. For each gene linked to an *EAR*_Endpoint_ threshold exceedance, the available gene annotation information was summarized to gain insight into potential specific biological functions and cellular responses that could be influenced. Gene ortholog information was obtained using the R package *homologene* (Mancarci & French, 2019). Information from DAVID was queried online using the R package *rDAVIDWebService* (Fresno & Fernandez, 2013). Associations of gene targets, biological pathway information in the PANTHER classification system, and mapping to AOPs for relevant ToxCast assays, chemicals, and chemical mixtures were determined using the R package *ToxMixtures* (Loken et al., 2021).

#### Watershed influences

Spearman correlation analysis was used to explore potential relations between TQ and EAR values and watershed attributes such as impervious surface, percentage of parking lot, land use, population density, and wastewater contribution. Land use attributes included six categories of urban; three categories of agriculture; mining and extraction; natural areas; water and wetlands; and aggregated urban and agricultural categories (Supporting Information, Table S1). Attributes related to wastewater contribution were annual wastewater effluent as a fraction of streamflow, and annual wastewater effluent as a fraction of streamflow weighted by the inverse of the distance upstream from the sampling location (Supporting Information, Table S1). Impervious surface, percentage of parking lot, land use attributes, and population density were determined using methods described elsewhere (Baldwin et al., 2020) and summarized in the Supporting Information. Wastewater contributions were determined using wastewater treatment plant (WWTP) discharge data (or permitted discharge when actual values were not available) and streamflow data for the period July 1, 2016–June 30, 2017, as described in Baldwin et al. (2016) and summarized in the Supporting Information. Spearman correlations were calculated using the R package *Hmisc* (Harrell et al., 2015), with a significance level (*p* value) of 0.05.

## RESULTS

### Chemical prioritization

#### Chemical occurrence

Of the 87 chemicals analyzed, 74 were detected at one or more locations (Supporting Information, Table S11 and Figure S1). The PAHs were the most frequently detected class of chemicals overall, with 32 of 36 PAHs detected at 90%–100% of the sites (Baldwin et al., 2020). Other chemicals with especially high detection frequencies were biphenyl (93%), anthraquinone (84%), carbazole (84%), 2,6‐dimethylnaphthalene (79%), indole (77%), 3‐methyl‐1H‐indole (77%), and *p*‐cresol (74%). Chemicals in the classes herbicides and insecticides were among the least frequently detected but are also relatively soluble, so they are not expected to have a substantial presence in sediment (with the exception of carbazole, which has numerous noninsecticide uses; see *Discussion*).

Chemicals in the classes PAH and sterols often occurred at the greatest concentrations, with median concentrations up to 496 and 2910 µg/kg, and maximum concentrations up to 39 900 and 25 200 µg/kg, respectively (Supporting Information, Table S11 and Figure S1). Indole, anthraquinone, carbazole, and *p*‐cresol also occurred at relatively high concentrations (median concentrations of 40–120 µg/kg).

The tendency of each particle to partition to sediment, as measured by *K*_OC_ values, appeared to influence chemical detection frequencies and concentrations. Chemicals with *K*_OC_ values greater than the median *K*_OC_ (greater than 3710) had a median detection frequency of 96% and a median concentration of 383 µg/kg, whereas chemicals with *K*_OC_ values less than the median *K*_OC_ had a median detection frequency of 7.1% and a median concentration of 117 µg/kg (Supporting Information, Figure S2).

All chemical concentrations and percentages of TOC in the sediments are provided in the Supporting Information, Table S12.

#### Potential biological effects from individual chemicals

**Figure 3:**
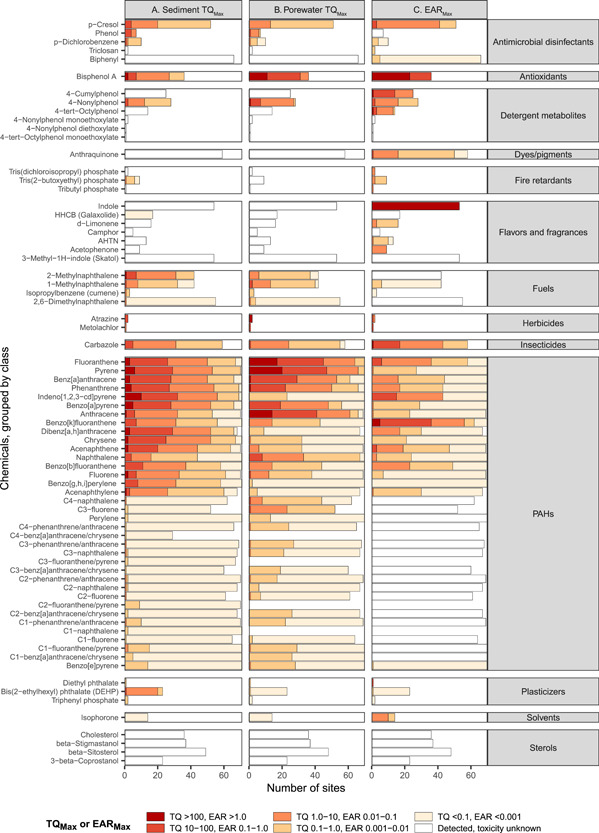
Summary of maximum (**A**) sediment and (**B**) porewater toxicity quotients (*TQ*_Max_), and (**C**) exposure–activity ratios (*EAR*_Max_) for organic chemicals measured in sediment samples from Great Lakes tributaries, 2017. Chemicals not detected are not shown (*n* = 13).

Sediment quality benchmarks were exceeded for 38 chemicals (sediment *TQ*_Max_ greater than 1.0; Figure 3A). The chemicals with the most frequent and/or greatest exceedances included many of the parent PAHs (including acenaphthene, phenanthrene, pyrene, indeno[1,2,3‐*cd*]pyrene, benz[*a*]anthracene, benzo[*a*]pyrene, fluoranthene, dibenz[*a,h*]anthracene, chrysene, fluorene, acenaphthylene, and anthracene), *p*‐cresol, bisphenol A, 4‐nonylphenol, 2‐methylnaphthalene, and carbazole. Most of these chemicals exceeded benchmarks at most sites and/or occurred at concentrations more than 10× the benchmark value (*TQ*_Max_ greater than 10). Estimated porewater concentration exceeded water quality benchmarks (*TQ*_Max_ greater than 1.0) for 32 chemicals, many of which were the same as those exceeding sediment quality benchmarks (Figure 3B). Porewater *TQ*_Max_ values were notably greater than sediment *TQ*_Max_ values for bisphenol A, 4‐nonylphenol, fluoranthene, pyrene, anthracene, and many of the alkylated PAHs. In contrast, sediment *TQ*_Max_ values were greater than porewater *TQ*_Max_ values for tris(2‐butoxyethyl) phosphate, hexahydro‐hexamethyl cyclopentabenzopyran, 2‐methylnaphthalene, indeno[1,2,3‐*cd*]pyrene, dibenz[*a,h*]anthracene, chrysene, acenaphthene, benzo[*ghi*]perylene, acenaphthylene, and di(2‐ethylhexyl)phthalate.

Estimated porewater concentration exceeded ToxCast screening values for 39 chemicals (*EAR*_Max_ greater than 0.001; Figure 3C). As with the sediment and porewater benchmarks, chemicals with the greatest EAR values included a number of the parent PAHs, *p*‐cresol, bisphenol A, 4‐nonylphenol, and carbazole. Several chemicals lacked known sediment or porewater benchmarks but exceeded ToxCast screening values, including biphenyl, 4‐cumylphenol, 4‐tert‐octylphenol, indole, anthraquinone, d‐limonene, and acetophenone. The *EAR*_Max_ values were especially high for two chemicals, indole and bisphenol A, exceeding 1.0 at many sites. On average, these two chemicals comprised 62.2% and 15.8%, respectively, of the *EAR*_Sample_ at each site (i.e., the sum of all *EAR*_Max_ values at each site). Overall, across all sites, at least one of the three benchmark types (sediment quality benchmarks, water quality benchmarks, and/or ToxCast screening values) was exceeded by 54 of the 87 chemicals, and 20 chemicals exceeded all three benchmark types.

**Figure 4:**
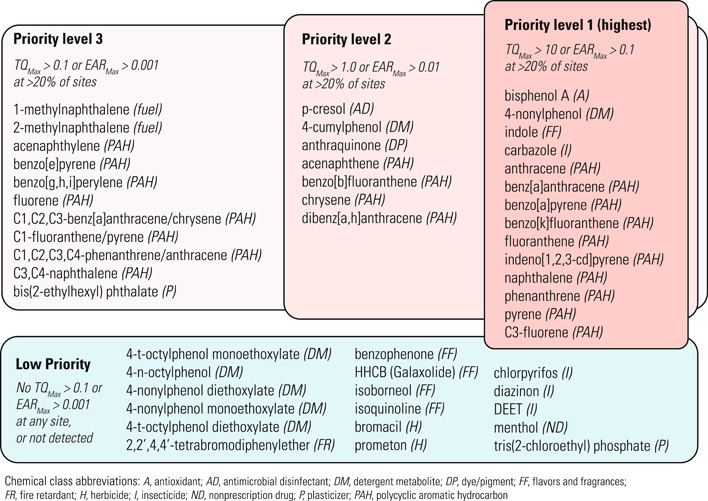
Prioritization of organic chemicals measured in sediment samples from Great Lakes tributaries in 2017 based on exceedance frequency and magnitude of maximum sediment or porewater toxicity quotients (*TQ*_Max_) or exposure–activity ratios (*EAR*_Max_). Chemicals not shown include those with detections but lacking benchmarks, and chemicals that exceeded a sediment or porewater TQ of 0.1 or EAR of 0.001 at 1%–20% of the sites.

Individual chemicals were prioritized based on the exceedance frequency and magnitude of sediment *TQ*_Max_, porewater *TQ*_Max_, or *EAR*_Max_ (Figure 4). The highest priority chemicals (Priority level 1) were those that exceeded a sediment or porewater *TQ*_Max_ of 10, or *EAR*_Max_ of 0.1, at more than 20% of the sites. Chemicals were identified as low priority if they did not exceed a sediment or porewater *TQ*_Max_ of 0.1 or *EAR*_Max_ of 0.001 at *any* site, or if they were not detected at any site. Chemicals that exceeded a sediment or porewater TQ of 0.1 or EAR of 0.001 at 1%–20% of the sites, and detected chemicals that lacked benchmarks, were not included in any prioritization category. The PAHs, which accounted for 41% of all chemicals analyzed, accounted for 71% of the Priority level 1 chemicals.

Potential bioeffects were unknown for a number of frequently detected chemicals because of a lack of sediment or water quality benchmarks and their absence in the ToxCast database. Most notably, 3‐methyl‐1H‐indole (skatol) was detected at 77% of the sites, but benchmarks for this chemical were not found. The sterols (3‐β‐sitosterol, β‐sitosterol, β‐stigmastanol, and cholesterol) also occurred frequently (33%–70% of the sites) but lacked data to calculate benchmarks.

### Site evaluation: occurrence and potential biological effects

**Figure 5:**
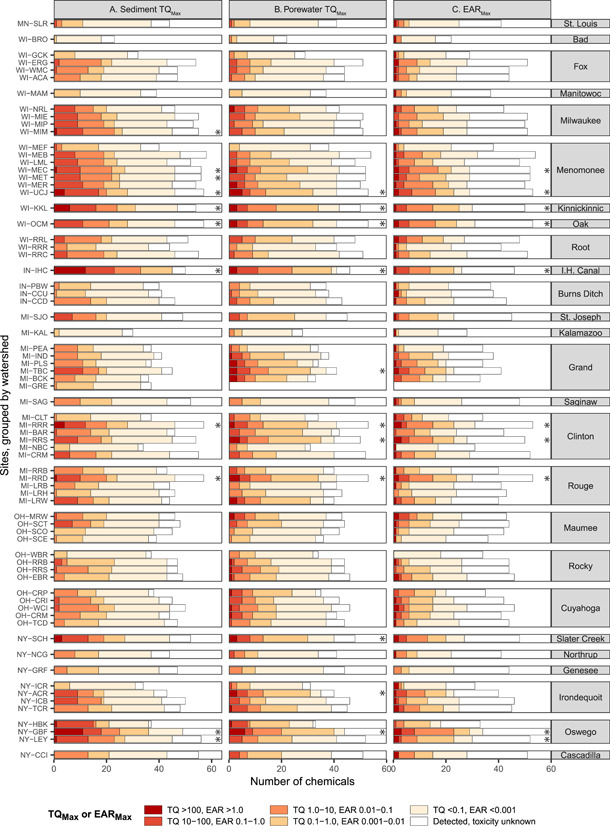
Summary of maximum (**A**) sediment and (**B**) porewater toxicity quotients (*TQ*_Max_), and (**C**) exposure–activity ratios (*EAR*_Max_) computed from organic chemical concentrations measured in sediment samples from Great Lakes tributaries, 2017. Sites are grouped by watershed and within each watershed are listed upstream to downstream, top to bottom. Site abbreviations are defined in Table 1. Asterisks mark the 10 sites with the greatest number of exceedances (sediment/porewater *TQ*_Max_ greater than 1.0, *EAR*_Max_ greater than 0.001) using each method.

A mixture of 23–64 chemicals was detected at each site (Figure 5 and Supporting Information, Figure S3 and Table S13). The site with the most chemicals detected was Geddes Brook at Fairmount, New York (NY‐GBF). Total sample concentrations ranged from 308 to 82 200 µg/kg except at three sites where concentrations were markedly greater: Indiana Harbor Canal at East Chicago, Indiana (IN‐IHC; 374 000 µg/kg), Geddes Brook (316 000 µg/kg), and Underwood Creek at Elm Grove, Wisconsin (WI‐UJC; 158 000 µg/kg). At Indiana Harbor Canal and Geddes Brook, the high total sample concentrations were primarily from PAHs (total PAH concentrations of 289 000 and 243 000 µg/kg, respectively). Underwood Creek had high concentrations of 4‐nonylphenol (51 600 µg/kg; nearly twofold greater than any other site) and bisphenol A (22 500 µg/kg; twofold greater than any other site), in addition to 57 700 µg/kg of ∑PAHs.

Sediment *TQ*_Max_, porewater *TQ*_Max_, and *EAR*_Max_ values for each site are summarized in Figure 5 and the Supporting Information, Table S13. The 10 sites with the most exceedances (sediment/porewater *TQ*_Max_ greater than  1.0, *EAR*_Max_ greater than  0.001) using each method are indicated with asterisks in Figure 5. Seven sites ranked in the top 10 using all three methods: Underwood Creek (WI‐UJC), Kinnickinnic River at Milwaukee, Wisconsin (WI‐KKL), Oak Creek at South Milwaukee, Wisconsin (WI‐OCM), Indiana Harbor Canal (IN‐IHC), Red Run at Warren, Michigan (MI‐RRR), River Rouge at Detroit, Michigan (MI‐RRD), and Geddes Brook (NY‐GBF). Site‐specific sediment *TQ*_Max_, porewater *TQ*_Max_, and *EAR*_Max_ values for individual chemicals are shown in the Supporting Information, Figures S4A–C and S5A–L.

### Potential bioeffects from chemical mixtures

#### PAH mixtures

Mixtures of PAHs exceeded the TEC at 44 sites (62%; median TECQ 1.6) and the PEC at 13 sites (18%; median PECQ 0.1; Supporting Information, Table S14 and Figure S6A and B; Baldwin et al., 2020). The PECQ values were greatest at Geddes Brook (NY‐GBF; PECQ 8.6) and at Indiana Harbor Canal (IN‐IHC; PECQ 5.9). The ∑ESBTU exceeded the threshold of 1.0 at 24 sites (38%; median ∑ESBTU 0.4; Supporting Information, Table S14 and Figure S6C; Baldwin et al., 2020). The sites with the greatest ∑ESBTU values were Geddes Brook (∑ESBTU 10.5), Tributary to Buck Creek Wyoming, Michigan (MI‐TBC; ∑ESBTU 5.2), and Lower River Rouge at Wayne Road at Wayne, Michigan (MI‐LRW; ∑ESBTU 5.1). The PEC and the ∑ESBTU were both exceeded at 11 sites (15%).

#### Alkylphenol mixtures

Alkylphenol mixtures at seven sites exceeded the alkylphenol toxicity quotient (*TQ*_AP_) of 1.0, indicating the potential for adverse biological effects on sensitive organisms (Canadian Council of Ministers of the Environment, 2002). The seven sites were River Rouge at Detroit (MI‐RRD; *TQ*_AP_ 1.2), Thomas Creek at East Rochester, New York (NY‐TCR; *TQ*_AP_ 1.2), Geddes Brook (NY‐GBF; *TQ*_AP_ 1.4), Slater Creek at Mount Read, New York (NY‐SCH; *TQ*_AP_ 2.3), Indiana Harbor Canal (IN‐IHC; *TQ*_AP_ 3.3), Oak Creek (WI‐OCM; *TQ*_AP_ 4.8), and Underwood Creek (WI‐UCJ; *TQ*_AP_ 17.7; Supporting Information, Figure S7). The high *TQ*_AP_ at Underwood Creek was primarily because of the 4‐nonylphenol concentration of 51 600 µg/kg.

#### EAR mixtures

Considering the chemical mixtures present in sediments, *EAR*_Endpoint_ calculations based on estimated porewater concentrations were used to help identify plausible biological targets or pathways influenced by the mixture. Overall, 22 ToxCast endpoints relating to nine gene targets exceeded the *EAR*_Endpoint_ threshold of 0.1 in at least 14 sites. Nine chemicals contributed to *EAR*_Endpoint_ at a level greater than EAR = 0.01 for these priority endpoints. Most chemicals were listed in Priority level 1 (Figure 4), with bisphenol A most frequently contributing to *EAR*_Endpoint_ threshold exceedances. The potential bioeffects of the priority endpoints include a variety of gene ontologies, biological functions, and pathways, which are explored further in the *Discussion* section.

## DISCUSSION

The present study used a variety of screening methods as a multiple‐lines‐of‐evidence approach to prioritize organic chemicals and sites in Great Lakes tributaries. Some chemicals and sites were repeatedly highlighted across different methods as having the potential to elicit biological effects. Others, however, were identified using only certain methods, resulting from the presence of a specific chemical or chemical class. Thus, although any of these methods can be used independently, combining them maximizes the number of chemicals screened, mitigates the uncertainties of individual methods, and strengthens common conclusions.

### Prioritization of sites

To help identify common conclusions from the different screening methods, results from the different methods were normalized into four priority levels (Table 3) and combined into a single table (Table 4). Definitions of priority levels were somewhat arbitrary but provide a consistent means of comparison across sites. Priority levels range from 1 to 4, with 1 indicating a high potential for adverse biological effects and 4 indicating no evidence for adverse biological effects. The overall priority score for each site was then calculated as the average across the different assessment methods, with a lower score indicating higher priority. Site priority scores are intended to provide a screening‐level assessment. Additional sampling should be done to verify chemical occurrence and/or adverse effects prior to any management actions based on these results. The highest priority sites were Geddes Brook at Fairmount, New York, Underwood Creek at Elm Grove, Wisconsin, Indiana Harbor Canal at East Chicago, Indiana, River Rouge at Detroit, Michigan, Red Run near Warren, Michigan, Kinnickinnic River at Milwaukee, Wisconsin, and Oak Creek at South Milwaukee, Wisconsin At each of these sites there were 20 or more chemicals exceeding a sediment *TQ*_Max_ of 1.0, 10 or more chemicals exceeding a porewater *TQ*_Max_ of 1.0, and 10 or more chemicals exceeding an *EAR*_Max_ of 0.01. In addition, the PAH mixture PECQ and/or ∑ESBTU threshold values of 1.0 were exceeded at each of these sites, and the alkylphenol mixture *TQ*_AP_ threshold value of 1.0 was exceeded at each site except for Red Run and the Kinnickinnic River. Thus, multiple lines of evidence indicate likely adverse effects on aquatic organisms at these sites.

**Table 3:** Criteria used for site prioritization

		Site priority level			
Name of bioeffects assessment	Criteria used for site prioritization	4 (lowest priority)	3	2	1 (highest priority)
Sediment *TQ*_Max_	Number of chemicals with *TQ*_Max_ > 1.0	0	1–9	10–19	≥20
Porewater *TQ*_Max_	Number of chemicals with *TQ*_Max_ > 1.0	0	1–9	10–19	≥20
*EAR*_Max_	Number of chemicals with *EAR*_Max_ > 0.01	0	1–9	10–19	≥20
PAH mixture	Exceedance of TECQ, PECQ, and/or ∑ESBTU	TECQ not exceeded	TECQ exceeded	PECQ or ∑ESBTU exceeded	PECQ and ∑ESBTU exceeded
Alkylphenol mixture	Value of *TQ*_AP_	0	0.01–0.99	1.0–9.9	≥10
Sediment *TQ*_Sample_	Value of sediment *TQ*_Sample_	1–9	10–99	100–999	1000–10 000
Porewater *TQ*_Sample_	Value of porewater *TQ*_Sample_	1–9	10–99	100–999	1000–10 000
*EAR*_Sample_	Value of *EAR*_Sample_	<0.1	0.1–0.99	1.0–9.9	≥10

*TQ*_Max_ = the maximum toxicity quotient for a given chemical in a sample; *EAR*_Max_ = the maximum exposure–activity ratio for a given chemical in a sample; PAH = polycyclic aromatic hydrocarbon; *TQ*_Sample_ = the sum of the *TQ*_Max_ values for all chemicals in a sample; *EAR*_Sample_ = the sum of the *EAR*_Max_ values for all chemicals in a sample; TECQ = consensus‐based threshold effect concentration quotient; PECQ = probable effect concentration quotient; ∑ESBTU = sum equilibrium partitioning sediment benchmark toxicity unit; *TQ*_AP_ = alkylphenol mixture toxicity quotient.

**Table 4:** Site prioritization summary based on different assessments of potential bioeffects of individual chemicals and mixtures of chemicals^a^

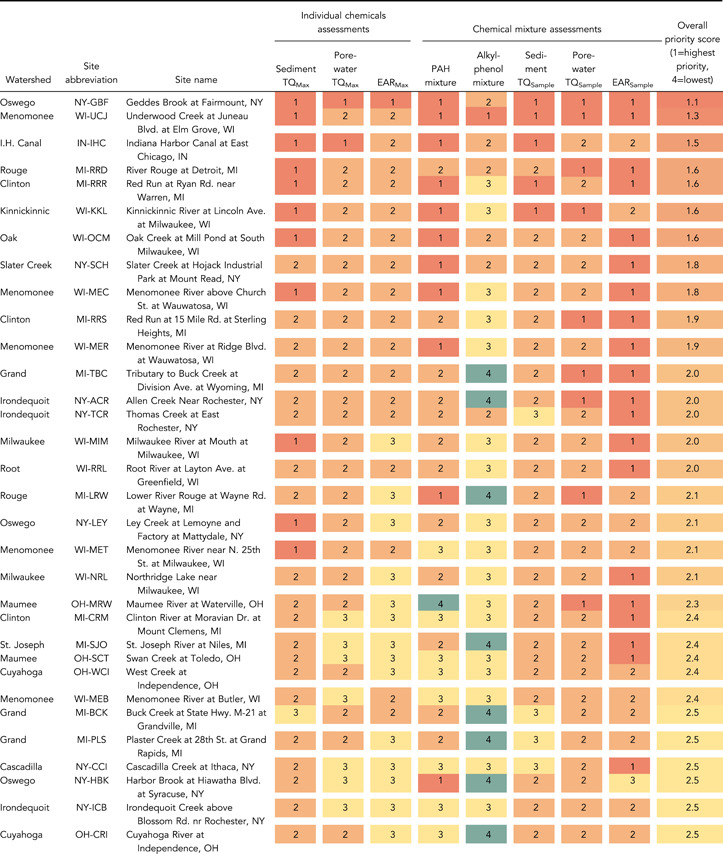											
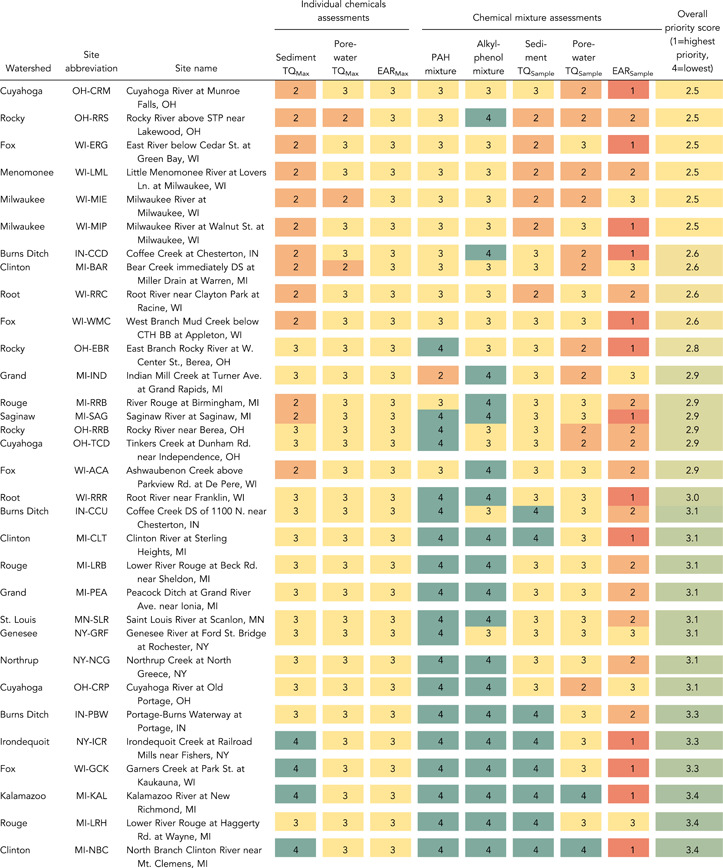											
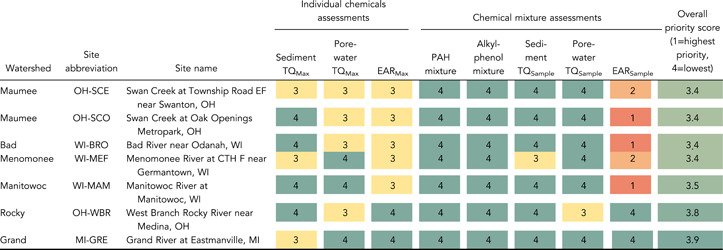											

^a^ The overall priority score is the average from all the methods, with a lower score indicating higher priority. Colors correspond to priority levels.

Abbreviations: *TQ*_Max_, the maximum TQ for a given chemical in a sample; *EAR*_Max_, the maximum exposure–activity ratio for a given chemical in a sample; PAH, polycyclic aromatic hydrocarbon; *TQ*_Sample_, the sum of the *TQ*_Max_ values for all chemicals in a sample; *EAR*_Sample_, the sum of the *EAR*_Max_ values for all chemicals in a sample; STP, sewage treatment plant; CTH, county trunk highway.

Several of the sites with high priority scores are located within Great Lakes Areas of Concern (AOCs), including Indiana Harbor Canal (Grand Calumet River AOC), River Rouge sites, Red Run (and other Clinton River sites), Kinnickinnic River, and some Menomonee and Milwaukee River sites (Milwaukee Estuary AOC; USEPA, 2021b). Beneficial Use Impairments common to many of these AOCs include degradation of fish and wildlife populations, fish tumors and other deformities, and degradation of phytoplankton and zooplankton populations, among others (USEPA, 2021b). The PAHs are among the primary contaminants identified at many of these AOCs, and our study found PAHs to be a primary driver of potential biological effects at these sites. However, many of these sites are also impacted by polychlorinated biphenyls, heavy metals, and other contaminants (USEPA, 2021b) that may also affect aquatic organisms.

Geddes Brook and Underwood Creek, the two sites with the highest priority scores, are not located within AOCs, nor, to our knowledge, do they have any other impairment designation (e.g., Superfund). Geddes Brook is most notable for having especially high PAH concentrations (*PAH*_16_ 196 000 µg/kg; greater than 18× the mean among other sites; Supporting Information, Table S14), likely associated with creosote and/or coal‐tar pavement sealant use (Baldwin et al., 2020) in the dominantly commercial/residential/suburban area upstream (Supporting Information, Table S1). Underwood Creek also had relatively high PAH concentrations (46 800 µg/kg; Supporting Information, Table S14) but is perhaps most notable for having a very high concentration of 4‐nonylphenol (56 000 µg/kg; greater than 26× the mean among other sites). The source of 4‐nonylphenol at Underwood Creek is not known.

Summed TQ values for each sample (i.e., site; sediment/porewater *TQ*_Sample_) were significantly correlated with numerous watershed attributes, the strongest among them being population density, parking lot area, urban commercial area, and urban and suburban area (*r* 0.52–0.66; Supporting Information, Table S15 and Figure S8). Summed EAR values for each sample (*EAR*_Sample_) were not significantly correlated with any watershed attributes, largely because of the disproportionate effect of indole on *EAR*_Sample_ values. Without indole, correlations of *EAR*_Sample_ values with watershed attributes were similar to those between sediment *TQ*_Sample_ values and watershed attributes. The fraction of streamflow as wastewater was not correlated or only poorly correlated with *TQ*_Sample_ and *EAR*_Sample_ (nor, for that matter, with *TQ*_Class_ and *EAR*_Class_), likely in part because 38 of the 71 sites had no direct wastewater contribution, and only 4 sites had greater than 10% wastewater contribution.

### Priority chemicals

The PAHs accounted for a large percentage of the chemicals prioritized in our study: PAHs represented 41% of the chemicals analyzed but 69% of the chemicals prioritized (Figure 4). The PEC and ∑ESBTU for PAH mixtures were exceeded at 18% and 38% of the sites, respectively (Supporting Information, Figure S6 and Table S14; Baldwin et al., 2020), indicating possible PAH‐related bioeffects at those sites. A prior exposure study using Milwaukee area stream sediments (including some of the same streams as the present study) demonstrated that exceedances of the PAH PEC and ∑ESBTU corresponded with significant immobility of *Hyalella azteca* in 91% and 85% of sediment samples, with increased mortality in 45% and 38% of sediment samples, respectively (Baldwin et al., 2017). The high concentration of PAHs in some Great Lakes tributaries relative to rivers and lakes in the western United States (Etheridge et al., 2014; Van Metre & Mahler, 2010; Yanagida et al., 2012) has been attributed primarily to coal‐tar–based pavement sealants (Baldwin et al., 2020). The PAH TQ and EAR values were significantly correlated with several watershed attributes, including impervious area, population density, parking lot area, and urban commercial area (Supporting Information, Figure S9 and Table S15).

Bisphenol A, classified as an antioxidant and a Priority level 1 chemical in the present study, is used in numerous industrial applications including the manufacture of plastics, paints, flame retardants, thermal papers, and brake fluids (Careghini et al., 2015; Zaugg et al., 2006). Bisphenol A has been shown to affect a number of endocrine‐related pathways, for example, both activating estrogen receptors and antagonizing androgen receptors in fish (see Ekman et al., 2012) and producing responses such as reductions in testosterone, sperm mobility and velocity, and increases in vitellogenin (Hatef et al., 2012). Some researchers have also reported that bisphenol A can cause epigenetic or transgenerational effects on endocrine endpoints in fish (Bhandari et al., 2015). In addition to endocrine disruption in fish, adverse effects of bisphenol A have been demonstrated on a variety of endpoints in a number of aquatic vertebrates and invertebrates (Brennan et al., 2006; Hirano et al., 2004). Pathways of bisphenol A to aquatic environments include industrial releases and WWTPs, among others (Flint et al., 2012). A study of WWTP biosolids from nine facilities across the United States reported bisphenol A concentrations up to 4600 µg/kg (Kinney et al., 2006). Two sites in the present study had concentrations considerably greater than the maximum reported in biosolids: Underwood Creek (22 500 µg/kg) and River Rouge at Detroit (11 000 µg/kg). These two sites are highly urban (80.8% and 92%, respectively) and do not receive WWTP effluent contributions, suggesting contamination from other urban sources such as industrial, commercial, or residential urban runoff or, in the case of River Rouge, combined sewer overflow discharges.

Indole, classified as a Priority level 1 chemical based on *EAR*_Max_ exceedances, has a variety of potential sources, both natural and anthropogenic. Indole is found in coal tar and tobacco smoke, is produced in the gut of animals and humans, and is therefore commonly used as a fecal indicator (Heberger et al., 2020). Despite its association with coal tar, indole was not significantly correlated with PAHs, nor with any urban‐related watershed attributes. Indole was most strongly correlated with naturally occurring chemicals including 3‐methyl‐1H‐indole (skatol; *r* = 0.88), β‐stigmastanol (*r* = 0.80), and β‐sitosterol (*r* = 0.78). Neither sediment nor porewater benchmarks were found for indole. The European Chemicals Agency (ECHA, 2021) reports a probable no‐effects concentration of 56.6 µg/kg in freshwater sediment, which is exceeded at 73% of the sites in the present study. Further evaluation of possible biological effects associated with the occurrence of indole in sediments may be warranted.

4‐Nonylphenol, classified as a Priority level 1 chemical in the present study, is a widely occurring alkylphenol primarily used as a raw material to make nonylphenol ethoxylates, which are used to make detergents and surfactants for a range of domestic and industrial purposes, including cleaning detergents, degreasers, food and beverage processing, textile and metal manufacturing, and plastic and paper production (Servos et al., 2003). In the environment, nonylphenol ethoxylates degrade to 4‐nonylphenol, which can persist in sediments for long periods of time, with a degradation half‐life of more than 60 years (Shang et al., 1999), and are known to be toxic (via narcosis) and disruptive to vertebrate endocrine pathways via activation of estrogen receptors (Servos et al., 2003). A review of 12 studies around the world reported no 4‐nonylphenol concentrations in excess of the sediment benchmark value of 1400 µg/kg (Chokwe et al., 2017), whereas in the present study 12 sites exceeded that benchmark. Concentrations of 4‐nonylphenol at Underwood Creek and Indiana Harbor Canal exceeded the benchmark by more than a factor of 10 (51 600 and 26 800 µg/kg, respectively). The *TQ*_AP_ values were primarily driven by 4‐nonylphenol, and were comparable to *TQ*_AP_ values in stormwater pond sediments in Minneapolis/St. Paul, Minnesota (Crane, 2019). An assessment of 237 chemicals in mussels around the Great Lakes reported that 4‐nonylphenol was one of only five chemicals, along with 4‐nonylphenol monoethoxylate and 4‐nonylphenol diethoxylate, present at all sampling locations (*n* = 32; Kimbrough et al., 2018).

Carbazole, classified as an insecticide and a Priority level 1 chemical in the present study, is a heterocyclic aromatic hydrocarbon that also is used in the manufacture of dyes, explosives, and lubricants (Zaugg et al., 2006), and occurs in tobacco smoke, coal tar, and coal‐tar–based soaps and oils used to treat psoriasis (carbazole is thought to give coal tar its antipsoriatic properties; Arbiser et al., 2006). Carbazole concentrations have previously been associated with urban land cover and/or wastewater contributions (Lee et al., 2005). In the present study, carbazole TQ and EAR values were significantly correlated with impervious area, population density, parking lot area, and urban commercial area, among other watershed attributes (Supporting Information, Figure S9 and Table S15). However, carbazole concentrations were more strongly correlated with total PAH concentrations (*r* = 0.77, *p* < 0.005) than with urban land cover (*r* = 0.57, *p* < 0.005) or any other watershed attributes analyzed, possibly suggesting a common source (Supporting Information, Figure S10A and B). The PAHs at most of the sites in the present study have been associated with coal‐tar–based pavement sealant (Baldwin et al., 2020); given that carbazole is a known component of coal tar, it is possible that coal tar is also a primary source of carbazole at these sites. Carbazole does not appear to be associated with wastewater, based on the poor relation to wastewater contribution (Supporting Information, Table S15) and the poor relation to concentrations of flavors and fragrances, a class of chemicals associated with wastewater (Supporting Information, Figure S10C).

Identified as a Priority level 2 chemical, *p*‐cresol is found in wood preservatives and creosote‐treated wood, pharmaceuticals, pesticides, and antioxidants (Heberger et al., 2020; Sullivan & Krieger, 2001; Zaugg et al., 2006), and is a metabolite of toluene, an industrial solvent (Kim et al., 1997). In a recent study, *p*‐cresol was associated with effects on the hepatic transcriptome in fathead minnows exposed in situ in wastewater‐effected streams (Schroeder et al., 2017). The greatest 15 concentrations of *p*‐cresol in the present study were within the range of concentrations reported in WWTP biosolids (Kinney et al., 2006).

Anthraquinone, identified as a Priority level 2 chemical, was frequently detected (at 84% of the sites) and appears to be related to PAHs and carbazole (Supporting Information, Figure S10D and G). Anthraquinone has multiple uses including in dye manufacturing and as a seed treatment and bird repellant (Zaugg et al., 2006) but is also a transformation product of the PAH anthracene (McKinney et al., 1999). Anthraquinone concentrations were better correlated with total PAH concentrations (*r* = 0.80, *p* < 0.005) and anthracene concentrations (*r* = 0.76, *p* < 0.005) than with urban land cover (*r* = 0.60, *p* < 0.005) or any other watershed attribute analyzed (Supporting Information, Figure S10D–F). Anthraquinone concentrations were also very well correlated with carbazole concentrations (*r* = 0.95, *p* < 0.005; Supporting Information, Figure S10G), possibly suggesting a common source. Anthraquinone concentrations were considerably greater than those reported in WWTP biosolids (maximum 217 µg/kg; Kinney et al., 2006), potentially eliminating WWTPs as the primary source. Despite its widespread occurrence, sediment screening benchmarks for anthraquinone were not found. The European Chemicals Agency (2020) reports a probable no‐effect concentration of 1414 µg/kg, derived from a single study.

Biphenyl, although not identified as a priority chemical, was frequently detected (at 93% of the sites). Biphenyl is used in the production of emulsifiers, dyes, and optical brighteners, as a pesticide, as a fungistat to preserve citrus fruits and other foods, and, historically, in the production of polychlorinated biphenyls (USEPA, 2011; Zaugg et al., 2006). In addition, biphenyl occurs naturally in crude oil, natural gas, and coal tar (USEPA, 2011). Like carbazole and anthraquinone, biphenyl concentrations were strongly correlated with total PAH concentrations (*r* = 0.81, *p* < 0.005; Supporting Information, Figure S10H), more so than with any other chemical class or watershed characteristic, including urban land cover (Supporting Information, Figure S10I).

3‐Methyl‐1H‐indole (skatol) was frequently detected (at 77% of the sites), but there were no data available to generate benchmarks. A weak aryl hydrocarbon receptor (AhR) agonist, 3‐methyl‐1H‐indole has been reported to induce cytochrome P450 1A (CYP1A) inhibition leading to cardiac deformities in zebrafish at a lowest‐observed‐effect water concentration (LOEC) of 13.1 µg/L (Brown et al., 2015). This LOEC is approximately equal to the highest estimated porewater concentration in the present study (13.4 µg/L, at Clinton River at Sterling Heights, Michigan). However, Brown et al. (2015) also reported significantly increased effects when 3‐methyl‐1H‐indole co‐occurred with fluoranthene (a CYP1A inhibitor), which was detected at all sites in the present study, creating a potential mixture of concern at some sites.

The sterols (cholesterol, 3‐β‐coprostanol, β‐sitosterol, and β‐stigmastanol) also occurred frequently but lack effects benchmarks or screening values. These chemicals occur naturally in human and animal waste (Martins et al., 2007). Despite their lack of benchmarks, these chemicals were not prioritized for additional study.

With the exception of carbazole, none of the chemicals included in the herbicides and insecticides classes were prioritized in the present study. The infrequent occurrence of these chemicals (atrazine, bromacil, metolachlor, prometon, chlorpyrifos, diazinon, and DEET) in sediment is to be expected, because most have low *K*_OC_ values or, in the case of chlorpyrifos and diazinon, have been regulated and seen reduced usage since the late 1990s and early 2000s (Stone et al., 2014). Thus, these chemicals do not provide a good representation of current‐use herbicides and insecticides that would be expected to accumulate in sediments.

### EAR mixtures and potential bioeffects

As a complement to identifying priority sites and contaminants, we also aimed to identify prominent biological activities and/or potential adverse effects that were associated with mixtures of chemicals that could act on common targets in an additive fashion, thereby contributing to the overall biological effects of mixtures detected. Although additive assumptions can be rather tenuous when comparing against a water quality benchmark based on apical effects, there is a stronger theoretical basis for assuming additivity of chemicals that are active in the same ToxCast assays. Thus, summed EARs for mixtures can readily be derived for specific ToxCast assays/targets (Blackwell et al., 2017). With this in mind, a combination of gene target functional annotations, AOPs, and other relevant literature was used to help contextualize the possible biological significance of pathway‐based activities of chemicals contributing to *EAR*_Endpoint_ greater than 0.1.

Mixtures of chemicals contributing to *EAR*_Endpoint_ greater than 0.1 at each site, and the related genes, ToxCast endpoints, and AOPs, are detailed in Supporting Information, Table S16. Several priority endpoints were related to genes involved in regulation of xenobiotic metabolism and excretion (Table 5). These endpoints included the AhR, pregnane X receptor (PXR; coded by NR1I2), and constitutive androstane receptor (CAR; coded by NR1I3), which are closely related transcription factors that regulate a variety of biological processes involved in xenobiotic metabolism (Willson & Kliewer, 2002). Bisphenol A, indole, carbazole, and four PAHs contributed to *EAR*_Endpoint_ threshold exceedances related to these endpoints. Activation of AhR and PXR were among the most frequently detected biological activities in a nationwide stream survey of the United States (Blackwell et al., 2019). Although these responses are sensitive, they may or may not be adverse in vivo depending on the severity (concentration) and duration of exposures and the life stage at which organisms are exposed. In many cases, activation of these pathways may lead to increased biotransformation and elimination of contaminants. However, AhR is an established molecular initiating event that can lead to early life stage mortality in fish that may have relevance in other oviparous vertebrates (AOPs 21 and 150; https://aopwiki.org). Analogs to CAR are generally absent in fish, so the significance of CAR to aquatic organisms is unclear. Evaluation of endpoints like CYP1A induction, cyclooxygenase‐2 and/or vascular endothelial growth factor expression, and embryo–larval toxicity in fish exposed to sediment porewater from sites with high *EAR*_Endpoint_ associated with ATG_AhR_Cis_up may be warranted. Markers of oxidative stress may also be worth exploring in exposed aquatic organisms.

**Table 5:** Gene descriptions and pathways for ToxCast endpoints with exposure activity ratios from chemical mixtures (*EAR*_*Endpoint*_) exceeding 0.1 in at least 14 sites (20%)^a^

Gene symbol	ToxCast endpoints	Chemicals	Gene effects, functional annotations, and biological pathways	Adverse outcome pathways (AOP nos.)
AhR	ATG_Ahr_CIS_up	Benz[*a*]anthracene	Aryl hydrocarbon receptor (AhR) is a transcription factor involved in the regulation of biological responses to planar aromatic hydrocarbons, including regulation of xenobiotic‐metabolizing enzymes such as cytochrome P450s	AhR activation leading to early life stage mortality in fish and birds (21, 150), hepatic steatosis (57), uroporphyria (131); sustained AhR activation leading to rodent liver tumors (41); AhR‐mediated epigenetic reproductive failure (310)
	TOX21_AhR_LUC_Agonist	Indeno[1,2,3‐*cd*]pyrene		
		Indole		
		Carbazole		
		Benzo[*b*]fluoranthene		
		Benzo[*k*]fluoranthene		
CYP2C11	NVS_ADME_rCYP2C11	Bisphenol A	Cytochrome P450s (CYPs) comprise a superfamily of monooxygenase enzymes, which catalyze many reactions involved in drug, steroid, and xenobiotic metabolism and synthesis of cholesterol, steroids, and other lipids	NA
ESR1 and ESR2	ACEA_ER_80hr	Bisphenol A	Estrogen receptor alpha (ESR1) and estrogen receptor beta (ESR2) are nuclear receptors essential for normal reproductive function, sexual differentiation and development in vertebrates	Estrogen receptor (ER) agonism leading to reproductive dysfunction (29), skewed sex ratios (52), and reduced survival (53); ER antagonism leading to reproductive dysfunction (30); modulation of adult Leydig cell function subsequent to estradiol activation in the fetal testis (67); increased dopaminergic activity leading to endometrial adenocarcinomas (112); anti‐estrogens and ovarian adenomas/granular cell tumors (165); early‐life ER activity leading to endometrial carcinoma in the mouse (167); ER activation leading to breast cancer (200)
	ATG_ERa_TRANS_up	4‐Cumylphenol		
	ATG_ERE_CIS_up	4‐Nonylphenol		
	NVS_NR_bER			
	NVS_NR_hER			
	OT_ERa_EREGFP_0120			
	OT_ERa_EREGFP_0480			
	TOX21_ERa_LUC_VM7_Agonist			
	OT_ER_ERaERb_0480			
	OT_ER_ERaERb_1440			
	OT_ER_ERbERb_0480			
	OT_ER_ERbERb_1440			
NR1I2 (PXR)	ATG_PXR_TRANS_up	Bisphenol A	Pregnane X receptor (PXR) is a nuclear receptor that regulates expression of proteins involved in xenobiotic metabolism and excretion	Nuclear receptor–induced thyroid hormone catabolism and developmental hearing loss (8); pentachlorophenol acute response by percellome (11); PXR activation leading to steatosis (60)
	ATG_PXRE_CIS_up	Indole		
NR1I3	NVS_NR_hCAR_Antagonist	Bisphenol A	Constitutive androstane receptor (CAR) is a nuclear receptor that regulates expression of proteins involved in xenobiotic metabolism and excretion	NR1I3 (CAR) suppression leading to hepatic steatosis (58); nuclear receptor–induced thyroid hormone catabolism and developmental hearing loss (8); CAR activation leading to hepatocellular tumors (107)
PPARA	NVS_NR_hPPARa	Bisphenol A	Peroxisome proliferator–activated receptor alpha (PPARα) is a ligand‐activated nuclear receptor and key regulator of lipid metabolism	PPARα antagonism leading to body‐weight loss (6); PPARα activation in utero leading to impaired fertility (18, 51); peroxisomal fatty acid β‐oxidation inhibition leading to steatosis (36); PPARα activation leading to liver tumors (37) and pancreatic acinar tumors (166); hepatic steatosis from NR1I3 (CAR) suppression (58), nuclear factor erythroid 2–related factor 2/farnesoid X receptor (NRF2/FXR) activation (61), and glucocorticoid receptor activation (318)
SLC6A2	NVS_TR_hNET	Carbazole	This solute carrier family 6 (SLC6) protein is a member of the sodium:neurotrasmitter symporter family, involved in regulation of norepinephrine homeostasis	NA
TPO	NCCT_TPO_AUR_dn	Bisphenol A	Thyroperoxidase (TPO) acts as an enzyme catalyzing thyroid hormone synthesis through iodination of thyroglobulins and coupling of iodotyrosyls to form thyroxine (T4)	TPO inhibition leading to adverse neurodevelopmental outcomes in mammals (42), increased mortality via reduced anterior swim bladder inflation (159), altered amphibian metamorphosis (175), impaired fertility in fish (271)
		Indole		

^a^Porewater chemical concentrations were estimated based on sediment concentrations collected in Great Lakes tributaries. Listed chemicals had an individual maximum exposure–activity ratio (*EAR*) of at least 0.01 in at least 14 sites. Gene effects, functional annotations, and pathways were summarized based on the Database for Annotation, Visualization and Integrated Discovery (DAVID; Huang et al., 2009; Laboratory for Human Retrovirology and Immunoinformatics, 2020), the Protein Analysis Through Evolutionary Relationships (PANTHER) classification system (Huaiyu et al., 2020; PANTHER, 2021), and AOP‐Wiki (Society for the Advancement of Adverse Outcome Pathways, 2018)

NA = not available.

A second group of priority endpoints reflects endocrine‐related activities. Most notably, 14 priority endpoints relate to estrogen receptor activity (Table 5) and were collectively linked to three chemicals (bisphenol A, 4‐cumylphenol, and 4‐nonylphenol). Bisphenol A has been broadly associated with endocrine disruption/toxicity (Ben‐Jonathan & Steinmetz, 1998; Vogel, 2009) through influences on multiple pathways, including activation of the estrogen receptor and antagonism of the androgen receptor (Ekman et al., 2012; Rubin, 2011). Estrogen is not only an important reproductive hormone, but also is involved in the neuroendocrine, skeletal, vascular, immune, and cardiovascular systems in humans (Hamilton et al., 2017; Lee et al., 2012). Agonism and antagonism of the estrogen receptor are listed as molecular initiating events in AOPs #29 and #30, respectively, leading to reproductive dysfunction in amphibians, birds, and/or fish (SAAOP, 2018). One additional priority endpoint (“NCCT_TPO_AUR_dn”) measures thyroid peroxidase (TPO) inhibition. As a critical enzyme involved in thyroid hormone production through conversion of iodide to iodine (Nelson et al., 2016; Stinckens et al., 2016), TPO can influence a number of physiological functions. Inhibition of TPO is linked to a number of AOPs (AOPs #42, #159, #178, and #271) and is included in a thyroid hormone disruption AOP network applicable to rodents, amphibians, and fish (Knapen et al., 2018; Stinckens et al., 2020). Bisphenol A and indole were linked to this endpoint, highlighting the potential influence of these chemicals on normal thyroid function.

Two additional endpoints (“NVS_TR_hNET” and “NVS_NR_hPPARa”) were prioritized based on the *EAR*_Endpoint_ results. The endpoint “NVS_TR_hNET” measures binding activity of a neurotransmitter transporter, specifically related to the SLC6A2 gene. The family of SLC6 genes mediates cross‐membrane transport of neurotransmitters, among other chemicals (Chen et al., 2004). For example, SLC6A2 is involved in norepinephrine homeostasis, which is found throughout chordates and is the primary neurotransmitter used by the sympathetic nervous system. Carbazole was linked to SLC6A2. Carbazole and other tricyclic chemicals, including several antidepressants, have long been known for their role in inhibiting norepinephrine uptake in mammals (Maxwell et al., 1969; Salama et al., 1971). Lastly, the endpoint, “NVS_NR_hPPARa” relates to peroxisome proliferator–activated receptor alpha (PPARα), a ligand‐activated nuclear receptor in vertebrates. The PPARs play a critical role in energy homeostasis and lipid metabolism and are also involved in cell proliferation and differentiation and in immune and inflammation responses (Pawlak et al., 2015). This endpoint was only linked to bisphenol A, highlighting the multiple potential biological pathways influenced by this single chemical.

### Considerations and limitations

The chemical and site prioritizations presented in our analysis are limited by the relatively small suite of chemicals analyzed. The chemicals included represent a diversity of chemical classes and uses but do not provide comprehensive coverage of current high‐use chemicals in the Great Lakes Basin. Important gaps include, but are not limited to, common current‐use pesticides that would be expected to accumulate in sediments, such as pyrethroids (Kuivila et al., 2012). Thus, although a conservative (i.e., protective) approach was used to assess the potential biological effects of the included chemicals, the likely presence of other chemicals may mean that potential biological effects are underestimated at some sites.

Porewater concentrations in the present study were derived from sediment concentrations and *K*_OC_ values (Equation 1). However, previous studies have shown that *K*_OC_ values can vary by orders of magnitude across different sediments based on the sorption properties of different types of carbon (i.e., natural organic carbon vs. black carbon; Hawthorne et al., 2006). Given this uncertainty, it should be emphasized that porewater concentrations are estimates. Similarities in the number and magnitude of exceedances between sediment and porewater benchmarks provide some confidence that porewater concentrations were not greatly over‐ or underestimated.

Chemical concentrations were not adjusted for laboratory recovery rates. For most chemicals, mean recovery rates were 80%–120% (Supporting Information, Table S3), but some chemicals had mean recovery rates of less than 60%, which biased the calculated TQ and EAR values low. Most notably, bisphenol A, identified as a Priority level 1 chemical, had a mean recovery rate of only 56%. Other chemicals with low recovery rates were infrequently detected (Supporting Information, Table S11). Conversely, TQ and EAR values are likely biased high for phenol, which had a mean recovery rate of 167% (Supporting Information, Table S3). Even so, phenol was not identified as a priority chemical.

In the present study we show how TQ and EAR values can be used as a screening tool for identifying chemicals with the potential to cause adverse biological effects. Exceedance of neither a TQ nor an EAR threshold necessarily means that adverse effects are occurring in the environment. The pathway descriptions noted in the *EAR mixtures and potential bioeffects* section offer guidance as to how detected chemicals conceptually could affect processes at the cellular level and the types of pathways that may be triggered, suppressed, and/or interrupted by organic chemicals in sediment. Further in vivo and in situ evidence is needed to verify that these effects are realized in environmental settings.

The present study does not account for all exposure and toxicokinetic factors that would influence toxicity. This is particularly true for sediment contaminants whose availability to the class of organisms for which the benchmarks were derived may be quite different in the laboratory than in the field. Likewise, although ToxCast assays cover a broad range of pathways, the assay compositions are mammalian‐centric. Although some highly conserved cellular stress response pathways are included, ToxCast does not necessarily cover all pathways that would be relevant to the biology of typical sediment‐dwelling organisms in aquatic systems (e.g., sediment invertebrates, aquatic macrophytes). There is also wide variability in the number and ranges of benchmarks and screening values for individual chemicals. Some chemicals had multiple benchmarks whereas others had few or none (Supporting Information, Figure S1). In addition, for those chemicals with multiple benchmarks, benchmark values often spanned orders of magnitude. As an example, seven sediment benchmarks were found for the PAH pyrene, ranging from 53 to 6970 µg/kg (Supporting Information, Table S5). The choice of which benchmark to use could therefore greatly impact the results. Our assessment used the lowest available benchmark for each chemical, to be conservative and minimize the occurrence of false negatives (Type 2 error), as is generally considered appropriate for screening‐level assessments.

Thus, these prioritization approaches should not be viewed as risk assessments. Instead, they provide an indication of relative potential and concern for adverse effects given the available lines of evidence. High priority designations are not a definitive determination of effects. Moreover, low priority designations do not guarantee that the chemical(s) in question provides no risk but indicate simply that there is no compelling evidence of adverse effects at this time. Considering the management goals for the specific Great Lakes tributaries, and reaches within the overall watersheds, these results simply aim to help resource managers, scientists, and risk assessors focus their limited resources on the chemicals, sites, and biological effects most likely to occur, based on the available evidence.

## Supporting Information

The Supporting information is available on the Wiley Online Library at https://doi.org/10.1002/etc.5286.

## Disclaimer

Any use of trade, product, or firm names is for descriptive purposes only and does not imply endorsement by the US Government. The views expressed in this article are those of the authors and do not necessarily represent the views or policies of the US Environmental Protection Agency. The US Environmental Protection Agency through the Office of Research and Development provided technical direction but did not collect, generate, evaluate, or use the environmental data described in the present study. This article has been peer reviewed and approved for publication consistent with US Geological Survey Fundamental Science Practices (https://pubs.usgs.gov/circ/1367/).

## Author Contributions Statement

Steven R. Corsi: Conception; study design; sample collection; technical review. Daniel L. Villeneuve: Conception; study design; technical review. Gerald T. Ankley: Conception; study design; technical review. Brett R. Blackwell: Conception; study design; technical review. Marc A. Mills: Conception; study design; sample collection. Peter L. Lenaker: Sample collection. Austin K. Baldwin: Data analysis; writing. Owen M. Stefaniak: Data analysis; writing. Luke C. Loken: Data analysis; writing. Michelle A. Nott: Geographic information system analysis.

## Supporting information

This article includes online‐only Supporting Information.

Supplementary information.

Supplementary information.

## Data Availability

Data are provided in the Supporting Information and are also available online at https://doi.org/10.5066/F7P55KJN (US Geological Survey 2021) and at https://waterdata.usgs.gov/nwis. Data, associated metadata, and calculation tools are also available from the corresponding author (akbaldwi@usgs.gov).
