# Supplementary material for: Risk‐Based Prioritization of Organic Chemicals and Locations of Ecological Concern in Sediment From Great Lakes Tributaries
Source: Environ Toxicol Chem. 2022 Feb 28;41(4):1016–41. doi: 10.1002/etc.5286 (PMC9306483; doi:10.1002/etc.5286)
Supplement: Supplementary file 2 — Supplementary information. [file ETC-41-1016-s002.docx]

Supplemental Information for

Risk-based prioritization of organic chemicals and locations of ecological concern in sediment from Great Lakes tributaries

Contents

[Sample collection 3](#_Toc94078074)

[Sample analysis 4](#_Toc94078075)

[Organic waste compounds 4](#_Toc94078076)

[Polycyclic aromatic hydrocarbons 4](#_Toc94078077)

[Total organic carbon 5](#_Toc94078078)

[Geographic information system methods 6](#_Toc94078079)

[ToxCast Results Included and Omitted 7](#_Toc94078080)

[**SI Figure S1.** Detected concentrations of chemicals in stream sediment samples collected from 71 Great Lakes tributaries, 2017, relative to sediment and water quality benchmarks and ToxCast activity concentration at cutoff (ACC) values. 8](#_Toc94078081)

[**SI Figure S2.** Relations between organic carbon-water partition coefficients (Koc) and (A) chemical detection frequency and (B) mean chemical concentration in stream sediment samples collected from 71 Great Lakes tributaries, 2017. Points represent individual chemicals. Mean chemical concentrations calculated using substitution of one-half the reporting level for results below the reporting limit. 9](#_Toc94078082)

[**SI Figure S3.** Number of compounds detected, total sample concentration, and percent of total concentration by class for each sampling location for stream sediment samples collected from Great Lakes tributaries, 2017. 10](#_Toc94078083)

[**SI Figure S4 A.** Maximum sediment toxicity quotients (TQMax) for each chemical at each site computed from organic waste compound concentrations measured in sediment samples from Great Lakes tributaries, 2017. 11](#_Toc94078084)

[**SI Figure S4 B.** Maximum porewater toxicity quotients (TQMax) for each chemical at each site computed from organic waste compound concentrations in porewater estimated from measured concentrations in sediment samples from Great Lakes tributaries, 2017. 12](#_Toc94078085)

[**SI Figure S4 C.** Maximum exposure activity ratios (EARMax) for each chemical at each site computed from organic waste compound concentrations in porewater estimated from measured concentrations in sediment samples from Great Lakes tributaries, 2017. 13](#_Toc94078086)

[**SI Figure S5 A.** Summary of maximum (A) sediment and (B) porewater toxicity quotients (TQMax), and (C) exposure-activity ratios (EARMax) computed from organic waste compound concentrations in the antimicrobial disinfectant class, measured in sediment samples from Great Lakes tributaries, 2017. 14](#_Toc94078087)

[**SI Figure S5 B.** Summary of maximum (A) sediment and (B) porewater toxicity quotients (TQMax), and (C) exposure-activity ratios (EARMax) computed from organic waste compound concentrations in the antioxidant class, measured in sediment samples from Great Lakes tributaries, 2017. 15](#_Toc94078088)

[**SI Figure S5 C.** Summary of maximum (A) sediment and (B) porewater toxicity quotients (TQMax), and (C) exposure-activity ratios (EARMax) computed from organic waste compound concentrations in the detergent metabolite class, measured in sediment samples from Great Lakes tributaries, 2017. 16](#_Toc94078089)

[**SI Figure S5 D.** Summary of maximum exposure-activity ratios (EARMax) computed from organic waste compound concentrations in the dye/pigment class, measured in sediment samples from Great Lakes tributaries, 2017. 17](#_Toc94078090)

[**SI Figure S5 E.** Summary of maximum (A) sediment toxicity quotients (TQMax) and (B) exposure-activity ratios (EARMax) computed from organic waste compound concentrations in the fire retardants class, measured in sediment samples from Great Lakes tributaries, 2017. 18](#_Toc94078091)

[**SI Figure S5 F.** Summary of maximum (A) sediment toxicity quotients (TQMax) and (B) exposure-activity ratios (EARMax) computed from organic waste compound concentrations in the flavors and fragrances class, measured in sediment samples from Great Lakes tributaries, 2017. 19](#_Toc94078092)

[**SI Figure S5 G.** Summary of maximum (A) sediment and (B) porewater toxicity quotients (TQMax), and (C) exposure-activity ratios (EARMax) computed from organic waste compound concentrations in the fuels class, measured in sediment samples from Great Lakes tributaries, 2017. 20](#_Toc94078093)

[**SI Figure S5 H.** Summary of maximum (A) sediment and (B) porewater toxicity quotients (TQMax), and (C) exposure-activity ratios (EARMax) computed from organic waste compound concentrations in the herbicides class, measured in sediment samples from Great Lakes tributaries, 2017. 21](#_Toc94078094)

[**SI Figure S5 I.** Summary of maximum (A) sediment and (B) porewater toxicity quotients (TQMax), and (C) exposure-activity ratios (EARMax) computed from organic waste compound concentrations in the insecticides class, measured in sediment samples from Great Lakes tributaries, 2017. 22](#_Toc94078095)

[**SI Figure S5 J.** Summary of maximum (A) sediment and (B) porewater toxicity quotients (TQMax), and (C) exposure-activity ratios (EARMax) computed from organic waste compound concentrations in the polycyclic aromatic hydrocarbons (PAHs) class, measured in sediment samples from Great Lakes tributaries, 2017. 23](#_Toc94078096)

[**SI Figure S5 K.** Summary of maximum (A) sediment and (B) porewater toxicity quotients (TQMax), and (C) exposure-activity ratios (EARMax) computed from organic waste compound concentrations in the plasticizers class, measured in sediment samples from Great Lakes tributaries, 2017. 24](#_Toc94078097)

[**SI Figure S5 L.** Summary of maximum (A) sediment and (B) porewater toxicity quotients (TQMax), and (C) exposure-activity ratios (EARMax) computed from organic waste compound concentrations in the solvents class, measured in sediment samples from Great Lakes tributaries, 2017. 25](#_Toc94078098)

[**SI Figure S6.** Potential toxicity of polycyclic aromatic hydrocarbon mixtures for stream sediment samples collected from Great Lakes tributaries, 2017 at each site using three assessment methods: (A) threshold effect concentration quotient (TECQ), (B) probable effect concentration quotient (PECQ), and (C) sum equilibrium partitioning sediment benchmark toxicity unit (ΣESBTU). 26](#_Toc94078099)

[**SI Figure S7.** Potential toxicity of the mixture of alkylphenols at each site for stream sediment samples collected from Great Lakes tributaries, 2017. 27](#_Toc94078100)

[**SI Figure S8.** Relations between select watershed attributes and potential biological effects summed for each sample for stream sediment samples collected from Great Lakes tributaries, 2017. 28](#_Toc94078101)

[**SI Figure S9.** Relations between select watershed attributes and potential biological effects summed for select organic chemical classes for stream sediment samples collected from Great Lakes tributaries, 2017. 29](#_Toc94078102)

[**SI Figure S10.** Relations between concentrations of individual chemicals (carbazole, anthraquinone, and biphenyl) and concentrations of other chemicals, summed concentrations of chemical classes, and watershed attributes for stream sediment samples collected from Great Lakes tributaries, 2017. 30](#_Toc94078103)

# Sample collection

Sediment sample collection was performed either by boat or while wading in the stream following USGS protocols (Shelton 2008). A WaterMark® Universal Core Head Sediment Sampler^[[1]](#footnote-1)^ (Forestry Suppliers, Jackson, MS) outfitted with a 2-3/4” outer diameter by 2-5/8” inner diameter with 1/16” wall polycarbonate tubing (Forestry Suppliers, Jackson, MS; or United States Plastic Corp®, Lima, OH) was used for sediment collection. To prepare polycarbonate tubing for sample collection it was cut to 18” in length, at 6.5” from the bottom of the 18” core tube a ¼” diameter hole was drilled. Polycarbonate tubes were then scrubbed with a brush using soapy water followed by three rinses each of tap water and deionized water prior to drying and bagging for transport to the sampling locations. An individual polycarbonate tube was prepared for each sampling location.

Prior to streambed sediment collection, electrical tape was used to cover the ¼” drill hole in the polycarbonate tube. Once applied, the core tube, utensils, and a stainless-steel pan were thoroughly rinsed with native sampling location water prior to initial contact with the sediment surface. Once a depositional location with fine grain sediment was identified, streambed sediment was collected by pushing the Universal Core Head Sampler with polycarbonate tubing into the sediment more than 6”. The core was carefully removed from the sediment and brought above the water surface, inspected to ensure that a good sediment core with the flocculant sediment-water interface was still intact, and excess bottom sediment was removed to a point where there was 6” of sediment left in the polycarbonate tube. Electrical tape was removed from the ¼” drill hole to allow the overlying water to drain without disruption to the sediment-water interface.

Sediment was removed from the polycarbonate tube directly into a stainless-steel pan while trying to maintain the sediment core structure. Using a stainless-steel spatula, the core was divided in half lengthwise from top to bottom, and each half was placed into separate 1 L amber glass jars. One jar was used for analysis of organic chemicals and the other was used for analysis of total organic carbon and an expanded suite of polycyclic aromatic hydrocarbons (PAHs). Samples were stored on ice for a maximum of 48 hours post collection until shipment to the laboratories. All sediment processing equipment (e.g., stainless-pan, spatulas, etc.) were field cleaned between each sampling location by scrubbing with soapy water followed by three rinses each of tap and deionized water.

# Sample analysis

## Organic waste compounds

Samples were analyzed for 51 organic waste compounds at the U.S. Geological Survey National Water Quality Laboratory (NWQL) in Denver, Colorado. The compounds were chosen because they are good indicators of domestic or industrial wastewater, and (or) because they are associated with human or environmental health risks (Kolpin et al. 2002; Zaugg et al. 2006). Compounds were extracted using a pressurized solvent-extraction system, then determined by capillary-column GC/MS (Burkhardt et al. 2006).

The NWQL spikes of these compounds in sand, river sediment, and topsoil show variable recovery performance. Percent recovery and variability data for each compound are listed in Zaugg and others (2006) and Burkhardt and others (2006). For most compounds, the NWQL sets the reporting limit (RL) at 2–10 times higher than the minimum detection limit (MDL; table 2; (Childress et al. 1999)). The RLs may be elevated because of interferences, especially in sediment samples. Reasons for interferences are discussed in Zaugg and others (2007) and Burkhardt and others (2006). Measured concentrations below the RL are still reported by the lab but are assigned an “estimated” (“E”) qualifier. The “E” qualifier also may be assigned if the concentration is below the lowest calibration standard [usually 0.2 micrograms per liter (μg/L)], or if there are matrix interferences. Further, some compounds have been permanently assigned an “E” qualifier because, historically, quality-assurance criteria have not been met. In terms of qualitative identification of a compound (detection as opposed to nondetection), results with the “E” qualifier generally can be considered reliable, with reliability decreasing as the measured concentration nears or falls below the MDL (Childress et al. 1999; Zaugg et al. 2006). In this report, concentrations reported with the “E” qualifier are considered detections.

## Polycyclic aromatic hydrocarbons

Methods for PAHs have previously been published and are reported here as well (Baldwin et al. 2020). Samples were analyzed for 18 parent and 18 alkylated PAHs by Battelle Memorial Institute in Norwell, Mass. (SI table S2). Upon their arrival at the laboratory, samples were stored refrigerated until homogenized and split for analysis. An aliquot was used for chemical analysis and was frozen. The remainder was refrigerated and forwarded to ALS Environmental, Kelso, Wash., for total organic carbon (TOC) analysis. Approximately 20 g of sediment was spiked with four surrogates and serially extracted three times with dichloromethane using an orbital shaker table. The combined extracts were concentrated by Kuderna-Danish and nitrogen evaporation techniques. Sample concentrates were further processed by alumina and copper cleanup, followed by size-exclusion high-performance liquid chromatography (HPLC) cleanup. Final extracts were fortified with internal standard (IS) compounds and submitted for PAH analysis by gas chromatography mass spectrometry (GC/MS). PAH analysis by GC/MS operated in selected ion monitoring (SIM) mode. An initial calibration of target analytes was analyzed prior to analysis to demonstrate the linear range of analysis. Calibration verification was performed at the beginning and end of each 24-hour period in which samples were analyzed. Concentrations of PAH were calculated versus internal standards using the average response factors (RF) generated from the initial calibration (EPA 8270D modified).

Four surrogate compounds were added to each environmental sample by Battelle prior to extraction: naphthalene-d8, acenaphthene-d10, phenanthrene-d10, and benzo(a)pyrene-d12. Recoveries of naphthalene-d8 ranged from 43 – 89% with a median of 59%. Similar recovery ranges and median values were noted for the remaining three compounds: 46 – 87%, median of 63% acenaphthene-d10; 40 – 88%, median of 60% benzo(a)pyrene-d12; and 46 – 94%, median of 69% phenanthrene-d10.

Battelle performed 11 procedural blanks, 5 matrix spikes, 5 lab duplicates, and 10 laboratory control spikes (LCS) over the course of five analytical batch analyses for this study. For the 11 procedural blank samples, there was a total of 396 PAH compound results from which 96% were below the detection limit. Of the 4% procedural blanks above the detection limit, only three compounds had exceedances: naphthalene (63%, 0.12 – 0.26 µg/kg), phenanthrene (31%, 0.08 – 0.15 µg/kg) and pyrene (6%, 0.12 µg/kg). All were flagged as “estimated” values. For the five matrix spikes, there were a total of 43 PAH compound results; percent recovery ranged from 44 – 117%, with a median of 73%. Battelle noted that “Several target analytes over all the analytical batch analyses had percent recoveries outside the measurement quality objective (MQO) range, however in each instance the concentration in the matrix spike was less than 5x the background concentration and therefore the results aren’t appropriate for data quality assessment.” Accordingly, there were no primary exceedances noted for the matrix spikes. The five lab duplicates’ relative percent difference (RDP) over all the compounds ranged from 0 to 197%, with a median of 26%. Over the course of the five analytical batches, there were 76 exceedances for duplicate precision, likely due to sample inhomogeneity. LCS percent recoveries ranged from 54 – 99%, median 79%.

## Total organic carbon

Methods for TOC have previously been published (Baldwin et al. 2020) and are reported here as well. TOC analysis was done using American Society for Testing and Materials (ASTM) D4129-05 modified method and a 0.05 percent reporting detection limit (RDL). All method blanks (n=5) were below the detection limit, and all duplicates (n=5) had relative percent differences < 4%. All samples for TOC analysis exceeded the recommended holding time, and the data were flagged appropriately.

# Geographic information system methods

Geographic information system (GIS) methods are described elsewhere (Baldwin et al. 2020). Briefly, watershed boundaries were determined using linework from the Watershed Boundary Dataset and catchments from the medium‐resolution NHDPlus V2 Dataset (U.S. Department of Agriculture-Natural Resources Conservation Service et al. 2009; U.S. Environmental Protection Agency and U.S. Geological Survey 2012). The National Water‐Quality Assessment (NAWQA) Area‐Characterization Toolbox (Price 2017) was used to calculate watershed percent imperviousness, parking lot abundance (both using the *Feature Statistics to Table* tool), and land‐use category percentages (using the *Tabulate Features to Percent* tool) (Falcone 2015; Homer et al. 2015; Falcone and Nott 2019). Watershed population density was calculated from 2010 census block data (U.S. Census Bureau Geography Division 2010).

Contributions from wastewater treatment plants (WWTPs) were estimated using methods described elsewhere (Baldwin et al. 2016). Briefly, wastewater treatment plant data were taken from the U.S. Environmental Protection Agency National Pollution Discharge Elimination System (NPDES) and Ontario Ministry of Natural Resources databases, with updates from either the International Joint Commission or the U.S. Geological Survey. Intersections of WWTP locations and sampling locations with NHDPlus catchment shapefiles were then used to characterize WWTPs within each site’s watershed and to calculate downstream distances.

# ToxCast Results Included and Omitted

ToxCast results with the following data quality flags were omitted from the current analysis: “Gain AC50 < lowest conc & loss AC50 < mean conc,” “Only highest conc above baseline, active,” “Biochemical assay with <50% efficacy,” and “Borderline active.” Tanguay_ZF_120hpf_ActivityScores were removed as these values were redundant with other zebrafish assays that appear individually in the ToxCast data. In addition, dose-response curves with poor responses or other atypical values resulted in the exclusion of 36 chemical-endpoint combinations, and three endpoints for all applicable chemicals (SI Table S7). The ToxCast assays ultimately included in the analysis, and their associated endpoints and targets, are listed in SI Table S8. The ToxEval input file with estimated porewater concentrations for each chemical at each site is provided in SI Table S9.

  
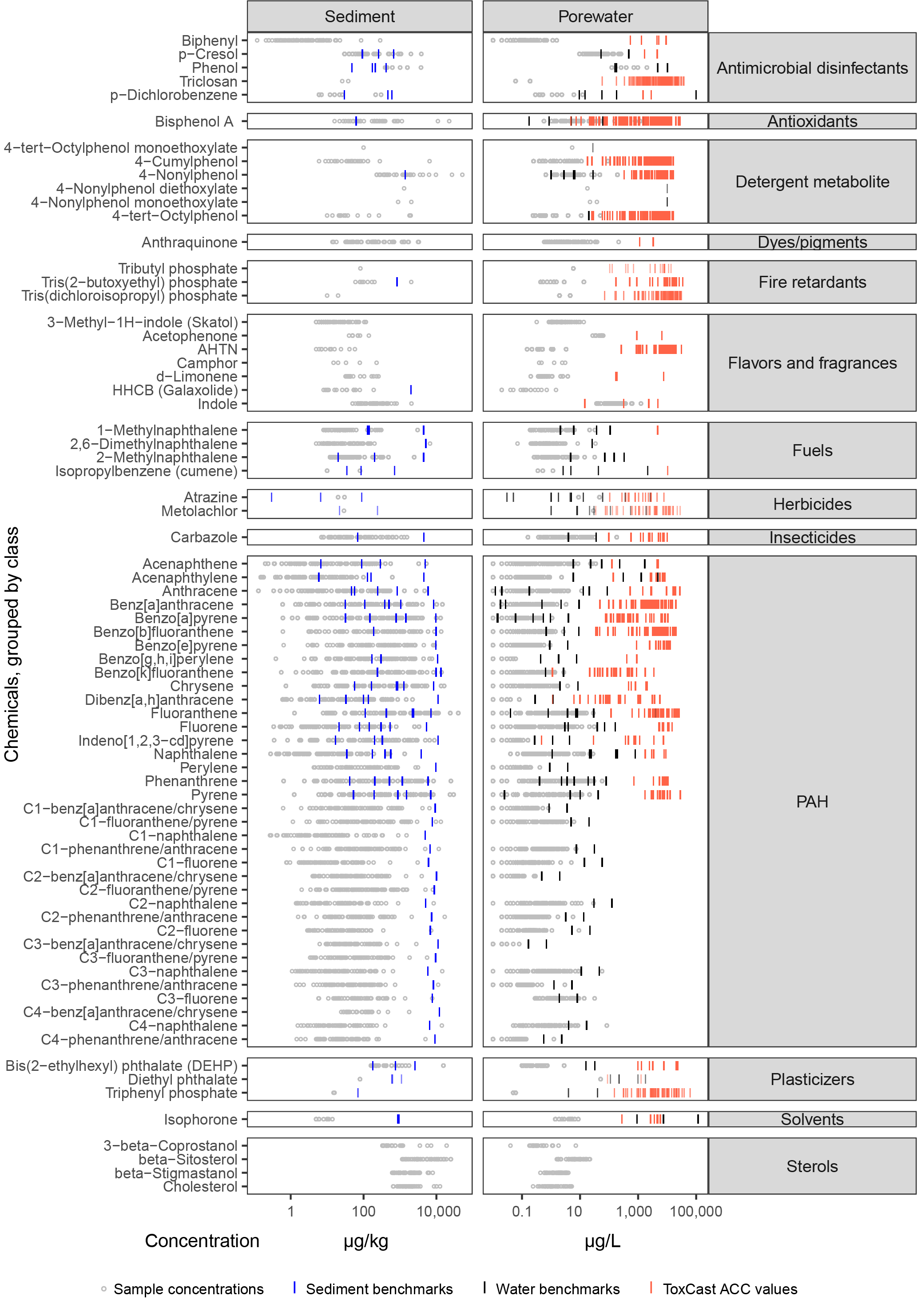


**SI Figure S1.** Detected concentrations of chemicals in stream sediment samples collected from 71 Great Lakes tributaries, 2017, relative to sediment and water quality benchmarks and ToxCast activity concentration at cutoff (ACC) values. Chemicals not detected are not shown. [PAHs, polycyclic aromatic hydrocarbons]


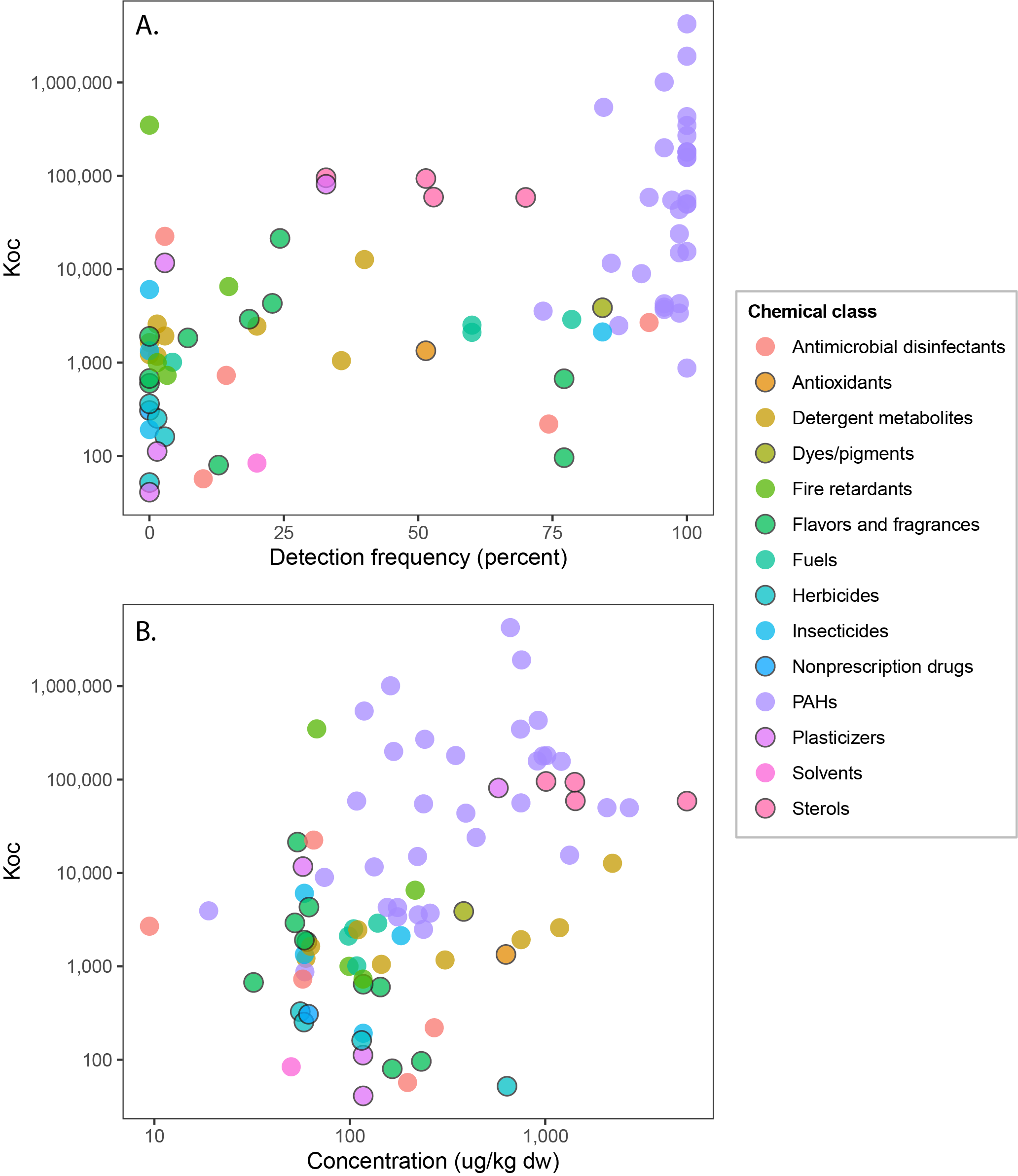


**SI Figure S2.** Relations between organic carbon-water partition coefficients (Koc) and (A) chemical detection frequency and (B) mean chemical concentration in stream sediment samples collected from 71 Great Lakes tributaries, 2017. Points represent individual chemicals. Mean chemical concentrations calculated using substitution of one-half the reporting level for results below the reporting limit.


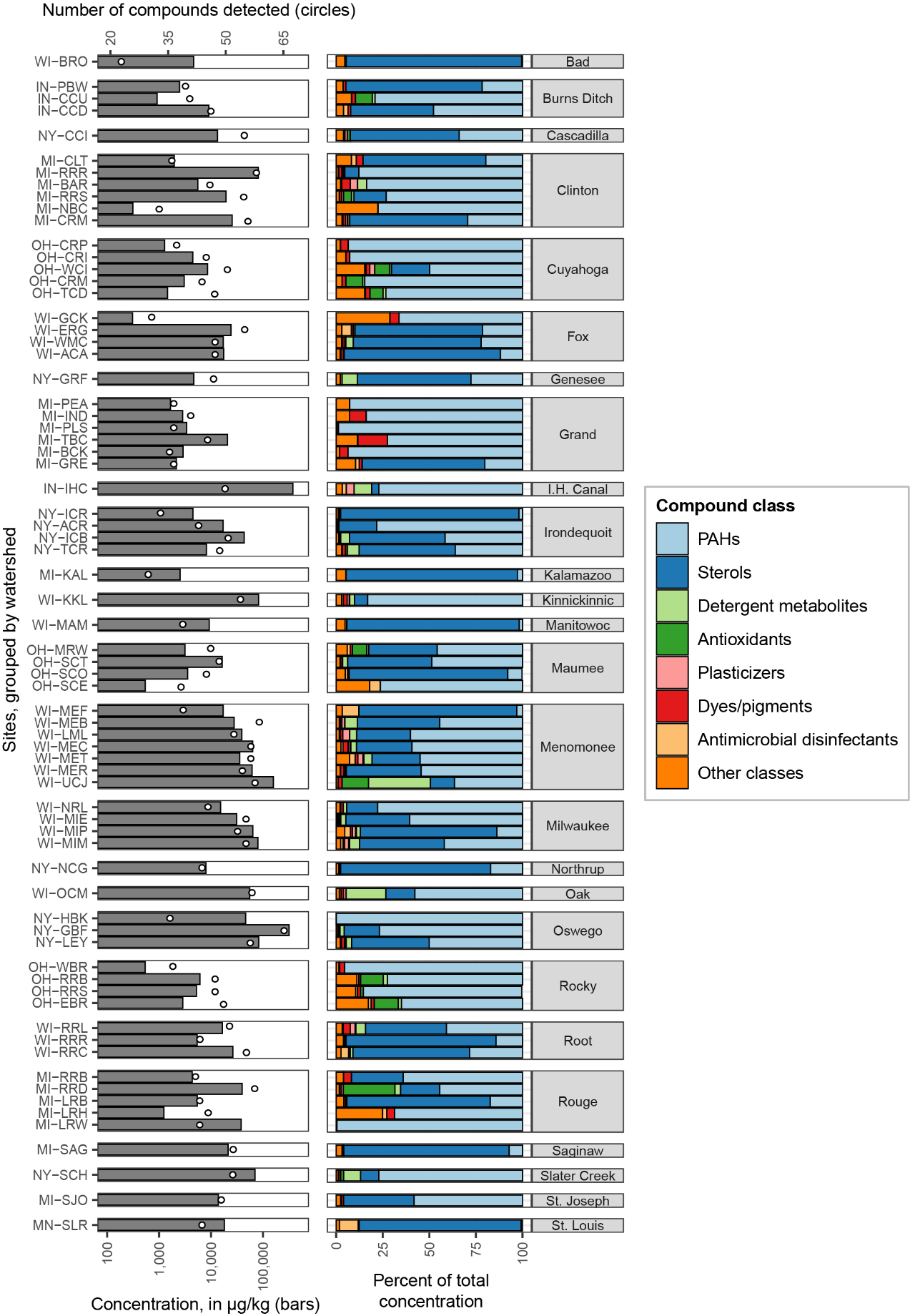


**SI Figure S3.** Number of compounds detected, total sample concentration, and percent of total concentration by class for each sampling location for stream sediment samples collected from Great Lakes tributaries, 2017. Site abbreviations are defined in Table 1 and SI Table S1. [PAHs, polycyclic aromatic hydrocarbons]


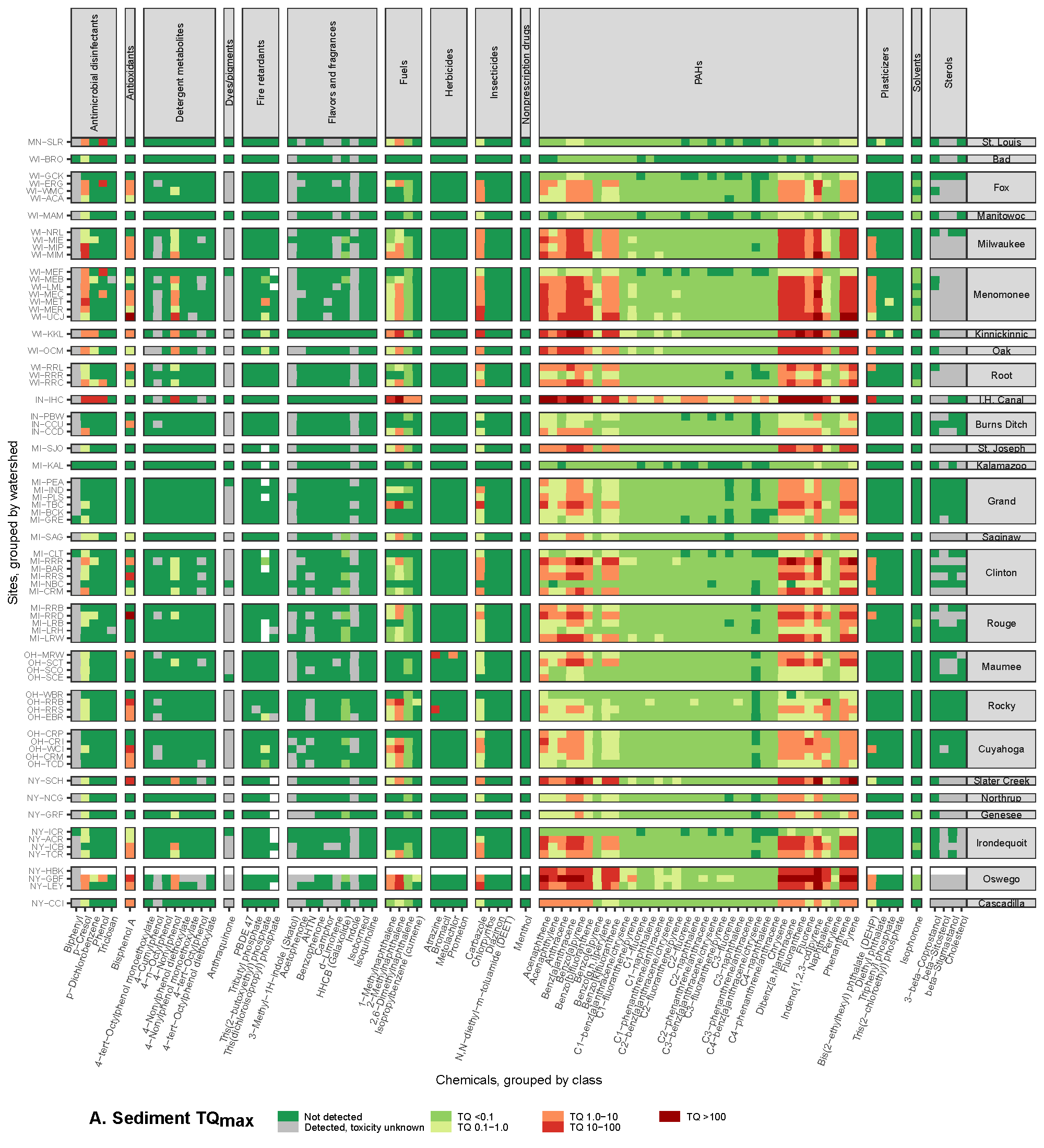


**SI Figure S4 A.** Maximum sediment toxicity quotients (TQMax) for each chemical at each site computed from organic waste compound concentrations measured in sediment samples from Great Lakes tributaries, 2017. Sites are grouped by watershed and within each watershed are listed upstream to downstream, top to bottom. Site abbreviations are defined in Table 1. [PAHs, polycyclic aromatic hydrocarbons]


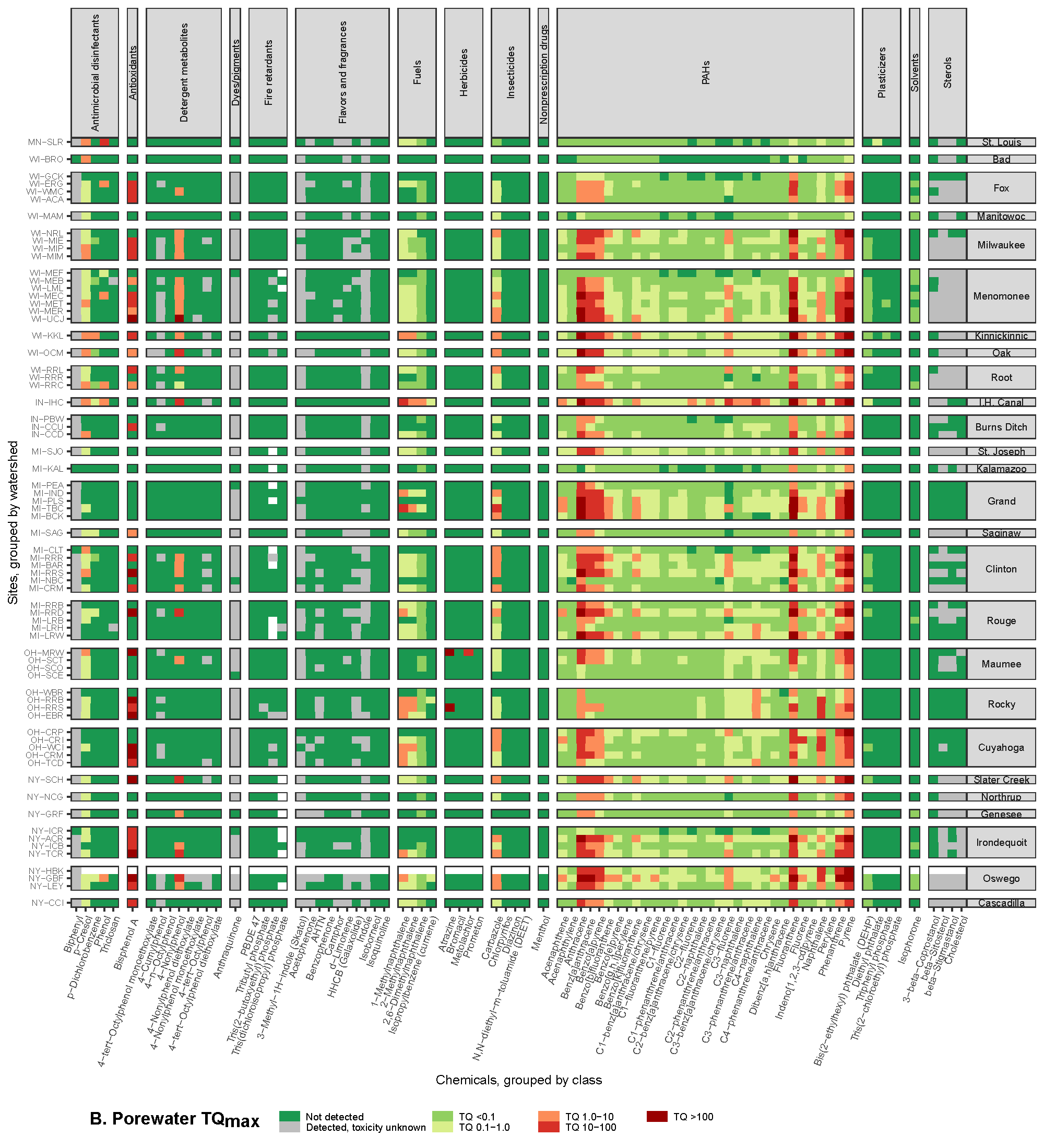


**SI Figure S4 B.** Maximum porewater toxicity quotients (TQMax) for each chemical at each site computed from organic waste compound concentrations in porewater estimated from measured concentrations in sediment samples from Great Lakes tributaries, 2017. Sites are grouped by watershed and within each watershed are listed upstream to downstream, top to bottom. Site abbreviations are defined in Table 1. [PAHs, polycyclic aromatic hydrocarbons]


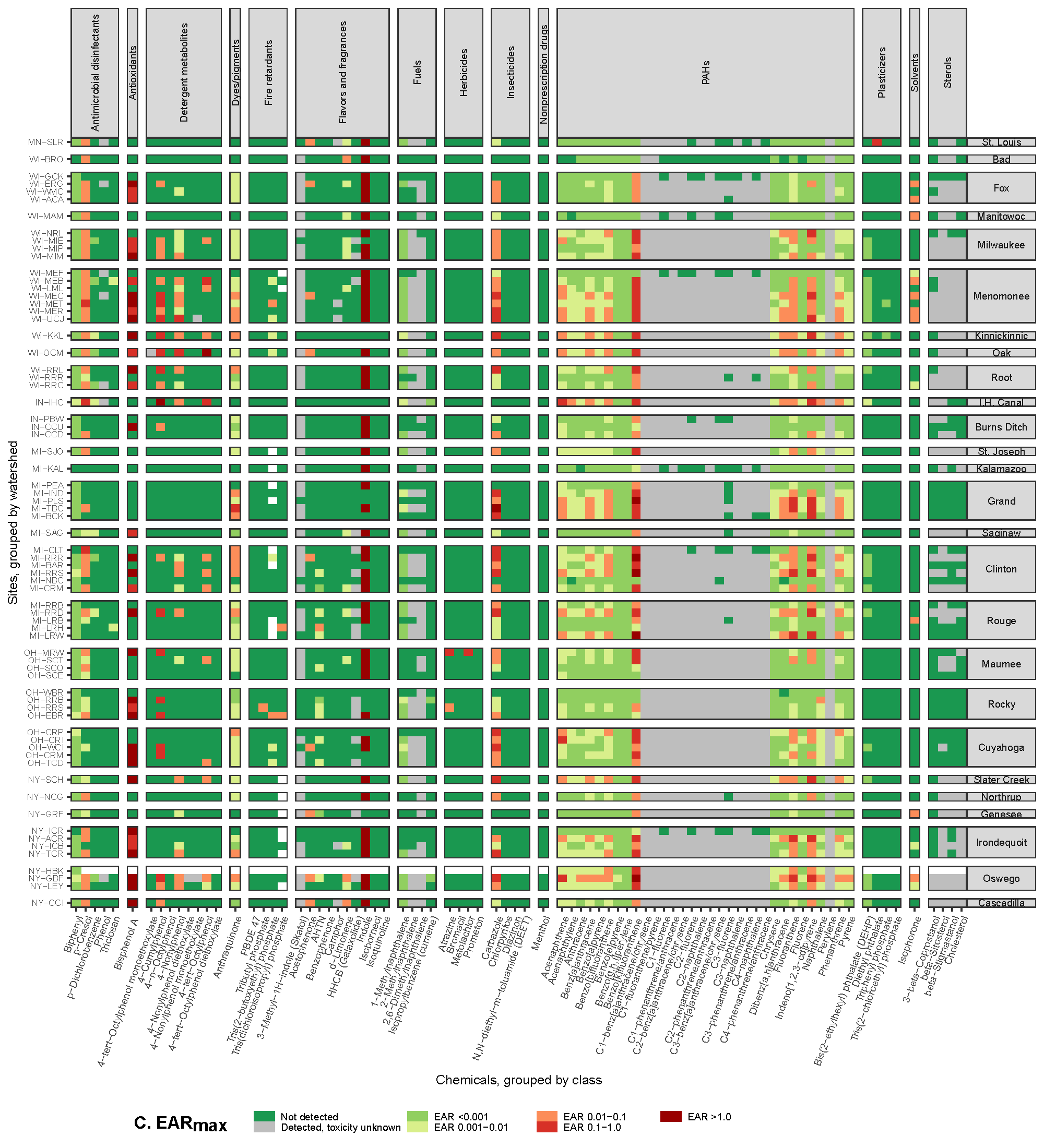


**SI Figure S4 C.** Maximum exposure activity ratios (EARMax) for each chemical at each site computed from organic waste compound concentrations in porewater estimated from measured concentrations in sediment samples from Great Lakes tributaries, 2017. Sites are grouped by watershed and within each watershed are listed upstream to downstream, top to bottom. Site abbreviations are defined in Table 1. [PAHs, polycyclic aromatic hydrocarbons]


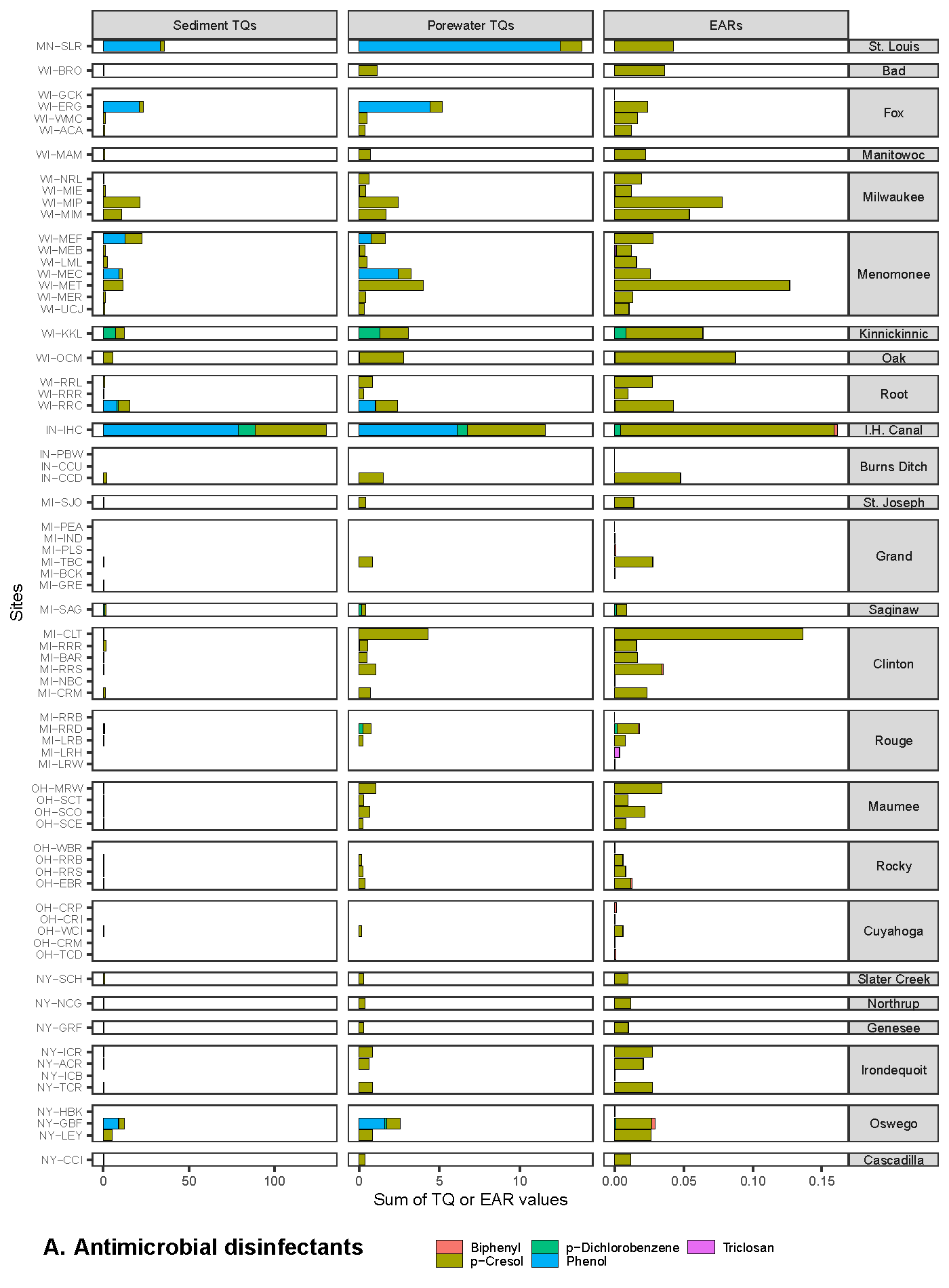


**SI Figure S5 A.** Summary of maximum (A) sediment and (B) porewater toxicity quotients (TQMax), and (C) exposure-activity ratios (EARMax) computed from organic waste compound concentrations in the antimicrobial disinfectant class, measured in sediment samples from Great Lakes tributaries, 2017. Sites are grouped by watershed and within each watershed are listed upstream to downstream, top to bottom. Site abbreviations are defined in Table 1.


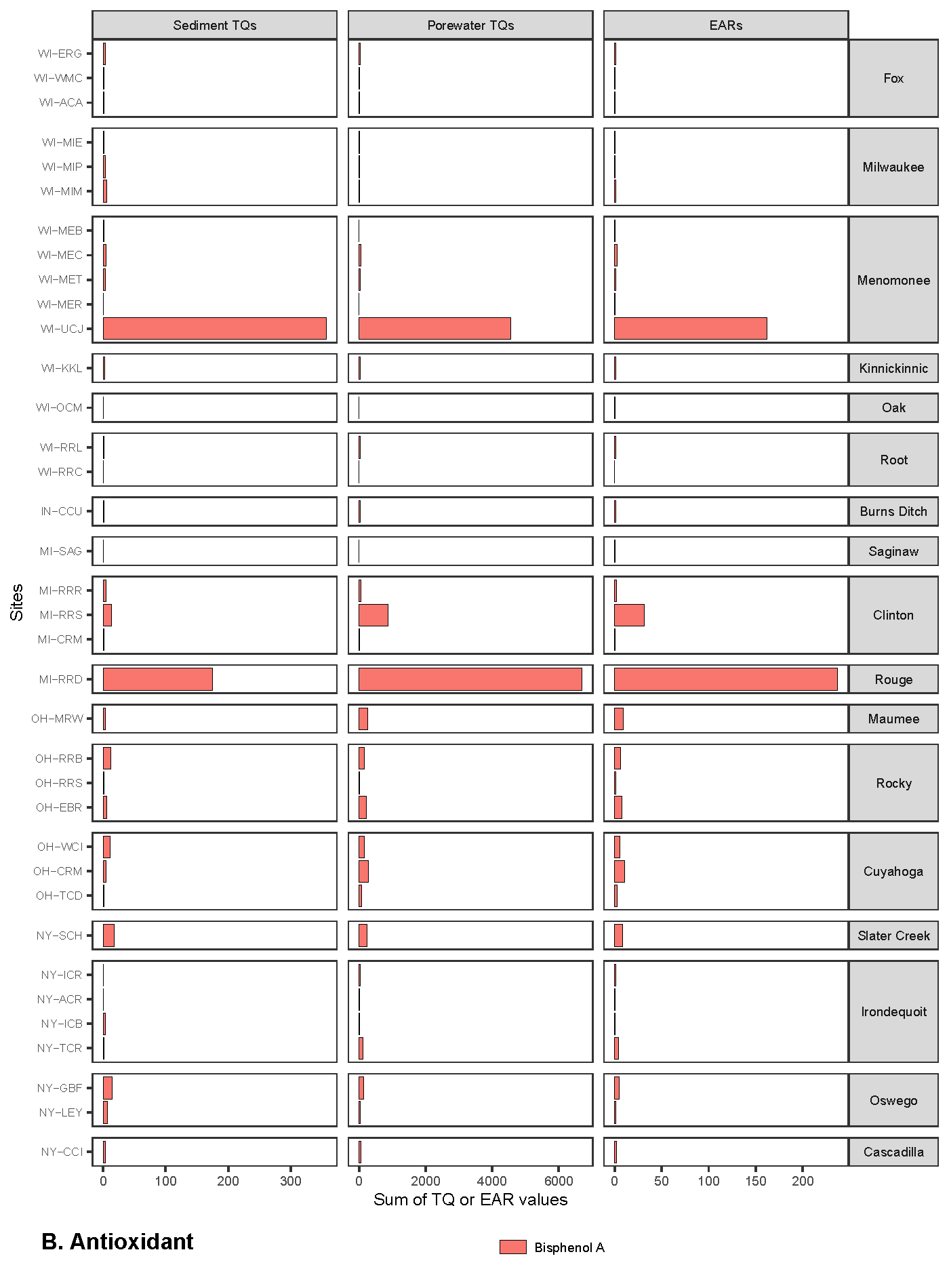


**SI Figure S5 B.** Summary of maximum (A) sediment and (B) porewater toxicity quotients (TQMax), and (C) exposure-activity ratios (EARMax) computed from organic waste compound concentrations in the antioxidant class, measured in sediment samples from Great Lakes tributaries, 2017. Sites are grouped by watershed and within each watershed are listed upstream to downstream, top to bottom. Site abbreviations are defined in Table 1.


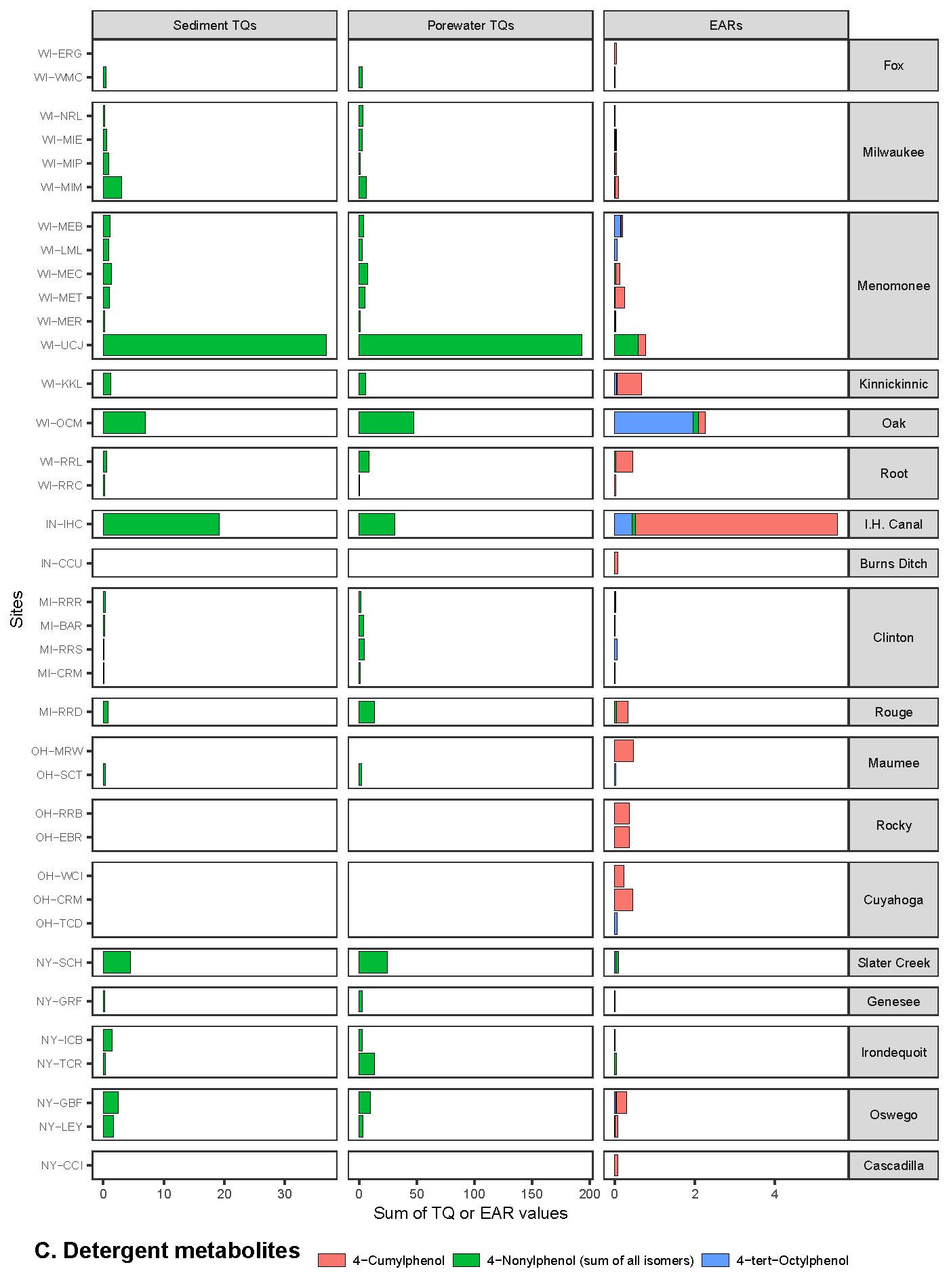


**SI Figure S5 C.** Summary of maximum (A) sediment and (B) porewater toxicity quotients (TQMax), and (C) exposure-activity ratios (EARMax) computed from organic waste compound concentrations in the detergent metabolite class, measured in sediment samples from Great Lakes tributaries, 2017. Sites are grouped by watershed and within each watershed are listed upstream to downstream, top to bottom. Site abbreviations are defined in Table 1.


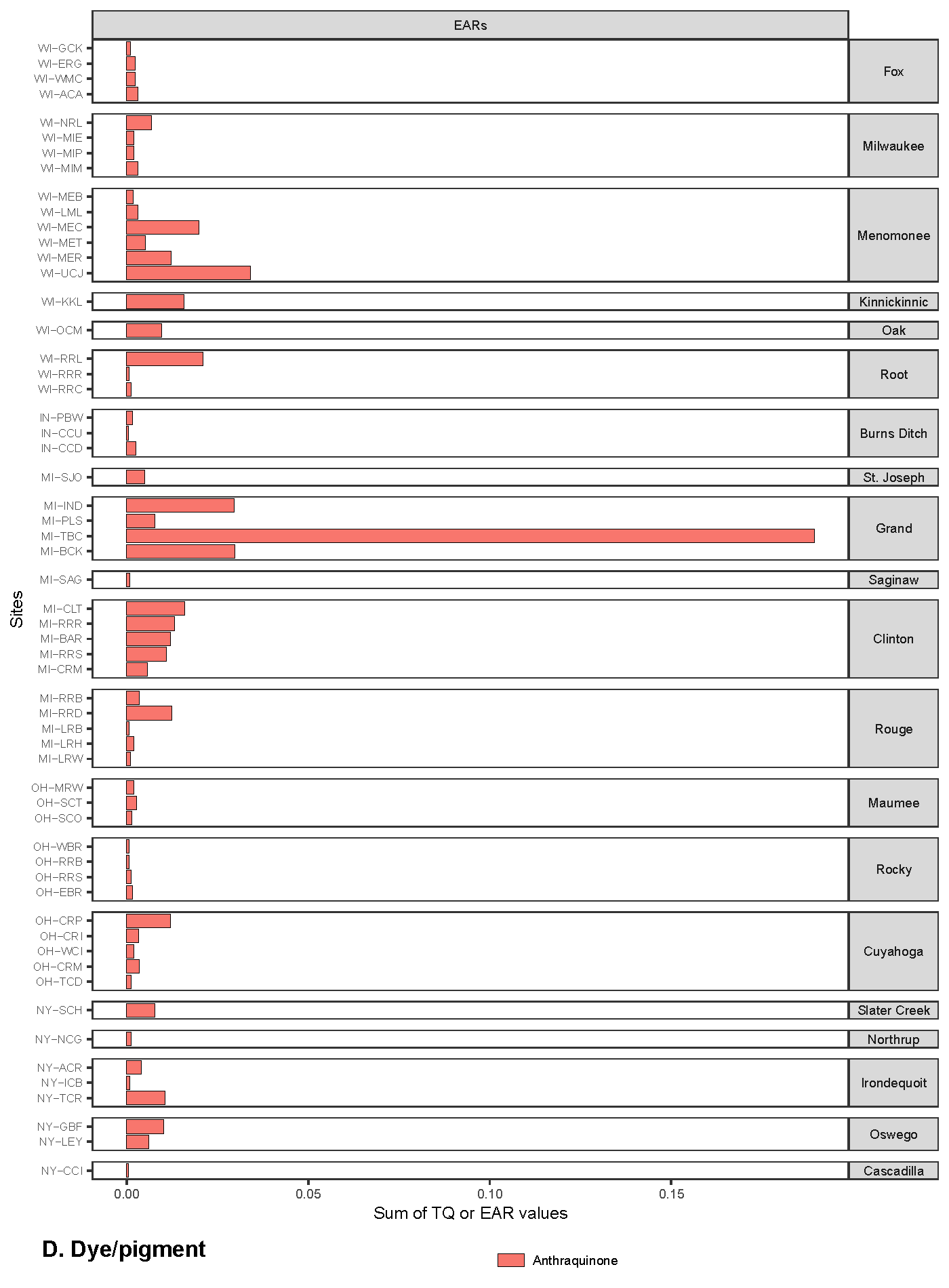


**SI Figure S5 D.** Summary of maximum exposure-activity ratios (EARMax) computed from organic waste compound concentrations in the dye/pigment class, measured in sediment samples from Great Lakes tributaries, 2017. Sites are grouped by watershed and within each watershed are listed upstream to downstream, top to bottom. Site abbreviations are defined in Table 1.


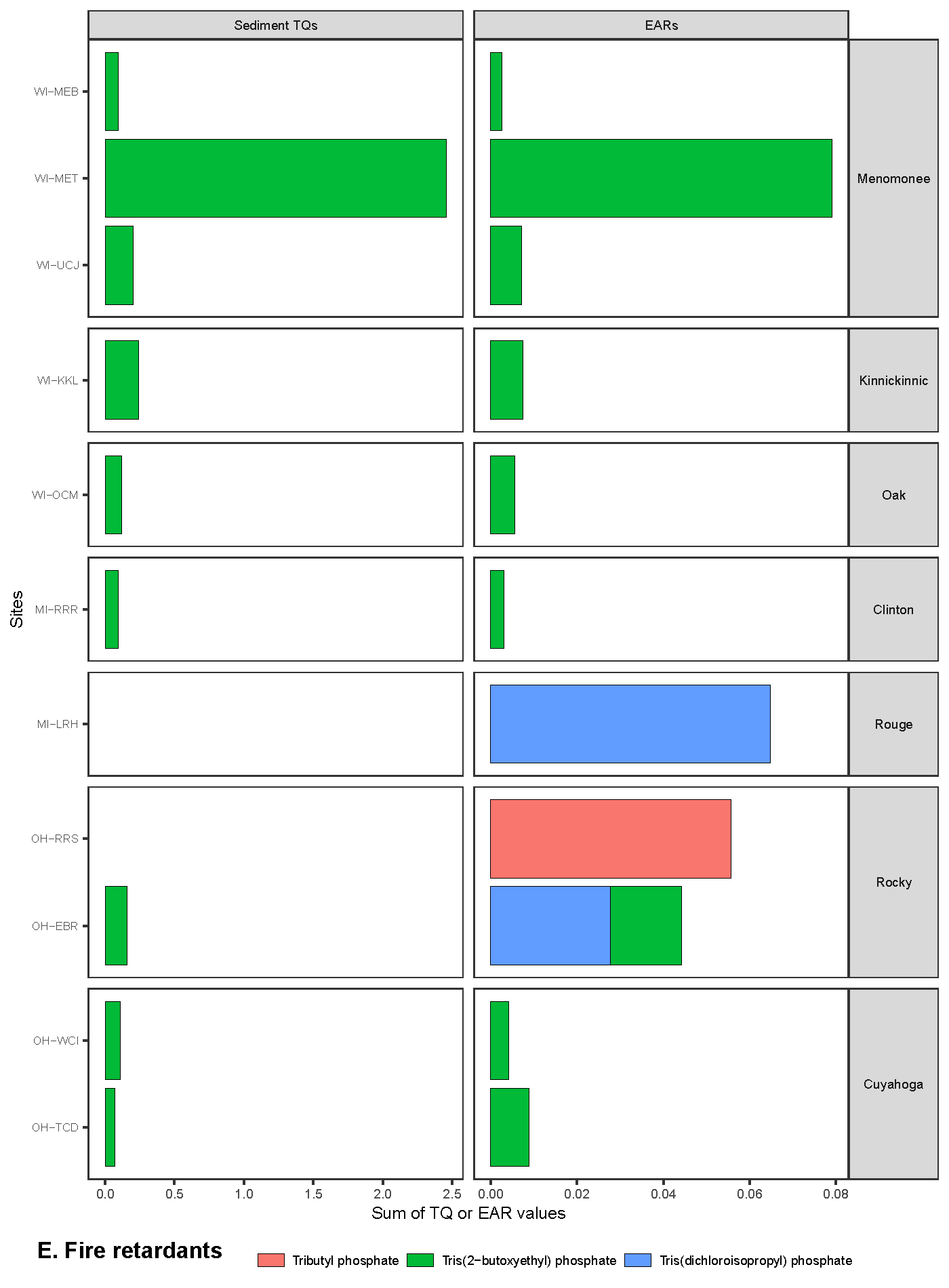


**SI Figure S5 E.** Summary of maximum (A) sediment toxicity quotients (TQMax) and (B) exposure-activity ratios (EARMax) computed from organic waste compound concentrations in the fire retardants class, measured in sediment samples from Great Lakes tributaries, 2017. Sites are grouped by watershed and within each watershed are listed upstream to downstream, top to bottom. Site abbreviations are defined in Table 1.


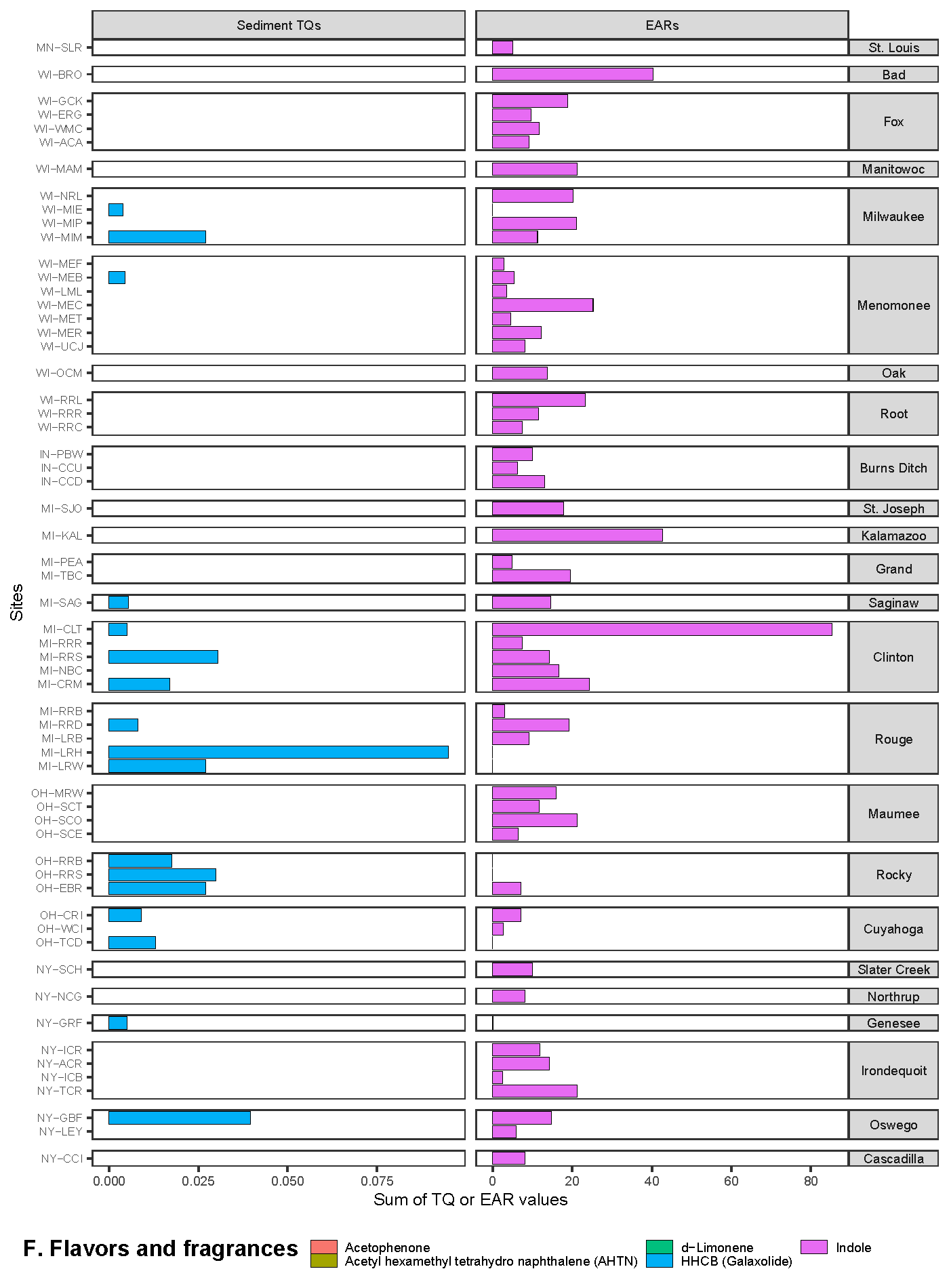


**SI Figure S5 F.** Summary of maximum (A) sediment toxicity quotients (TQMax) and (B) exposure-activity ratios (EARMax) computed from organic waste compound concentrations in the flavors and fragrances class, measured in sediment samples from Great Lakes tributaries, 2017. Sites are grouped by watershed and within each watershed are listed upstream to downstream, top to bottom. Site abbreviations are defined in Table 1.


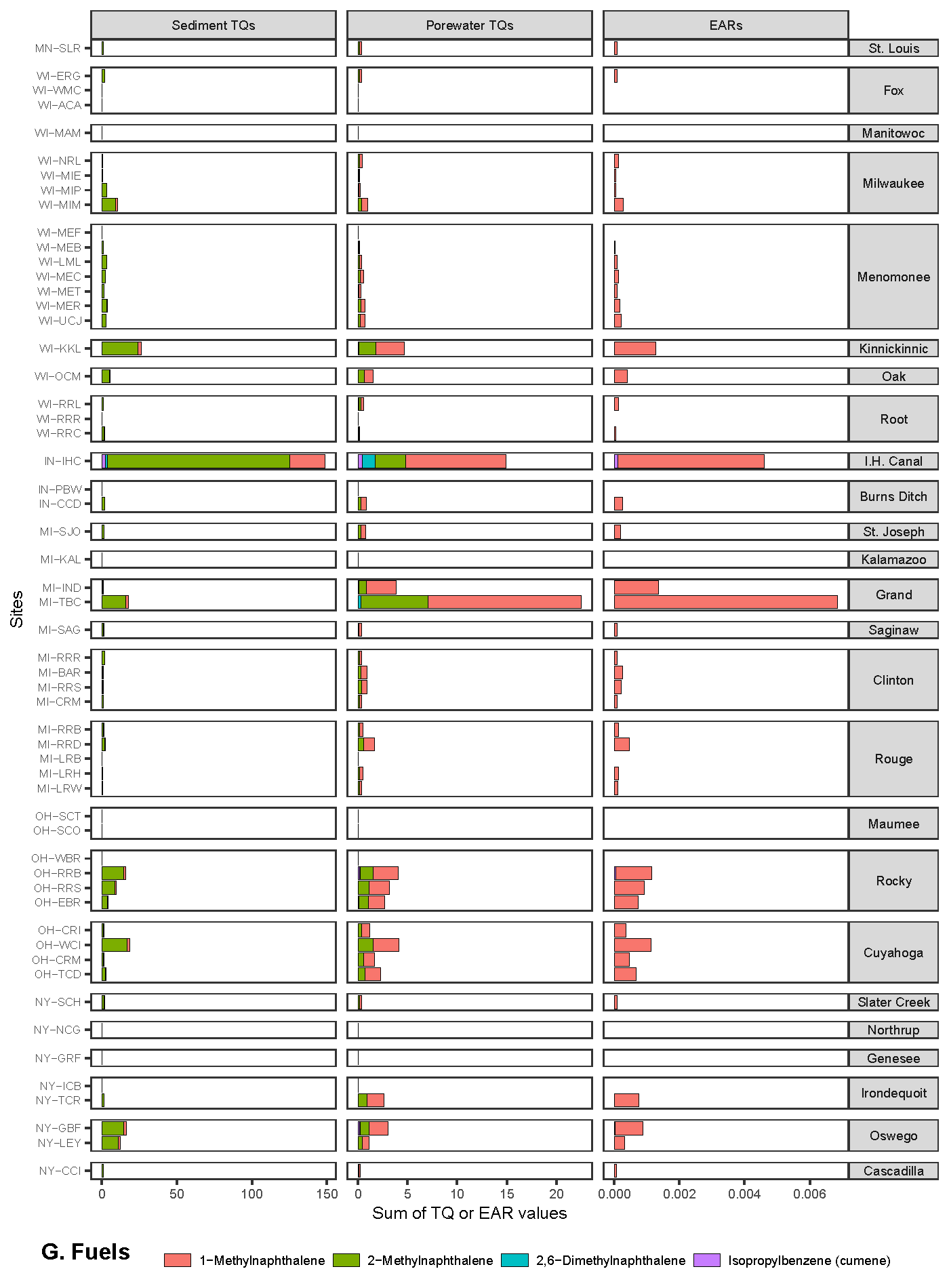


**SI Figure S5 G.** Summary of maximum (A) sediment and (B) porewater toxicity quotients (TQMax), and (C) exposure-activity ratios (EARMax) computed from organic waste compound concentrations in the fuels class, measured in sediment samples from Great Lakes tributaries, 2017. Sites are grouped by watershed and within each watershed are listed upstream to downstream, top to bottom. Site abbreviations are defined in Table 1.


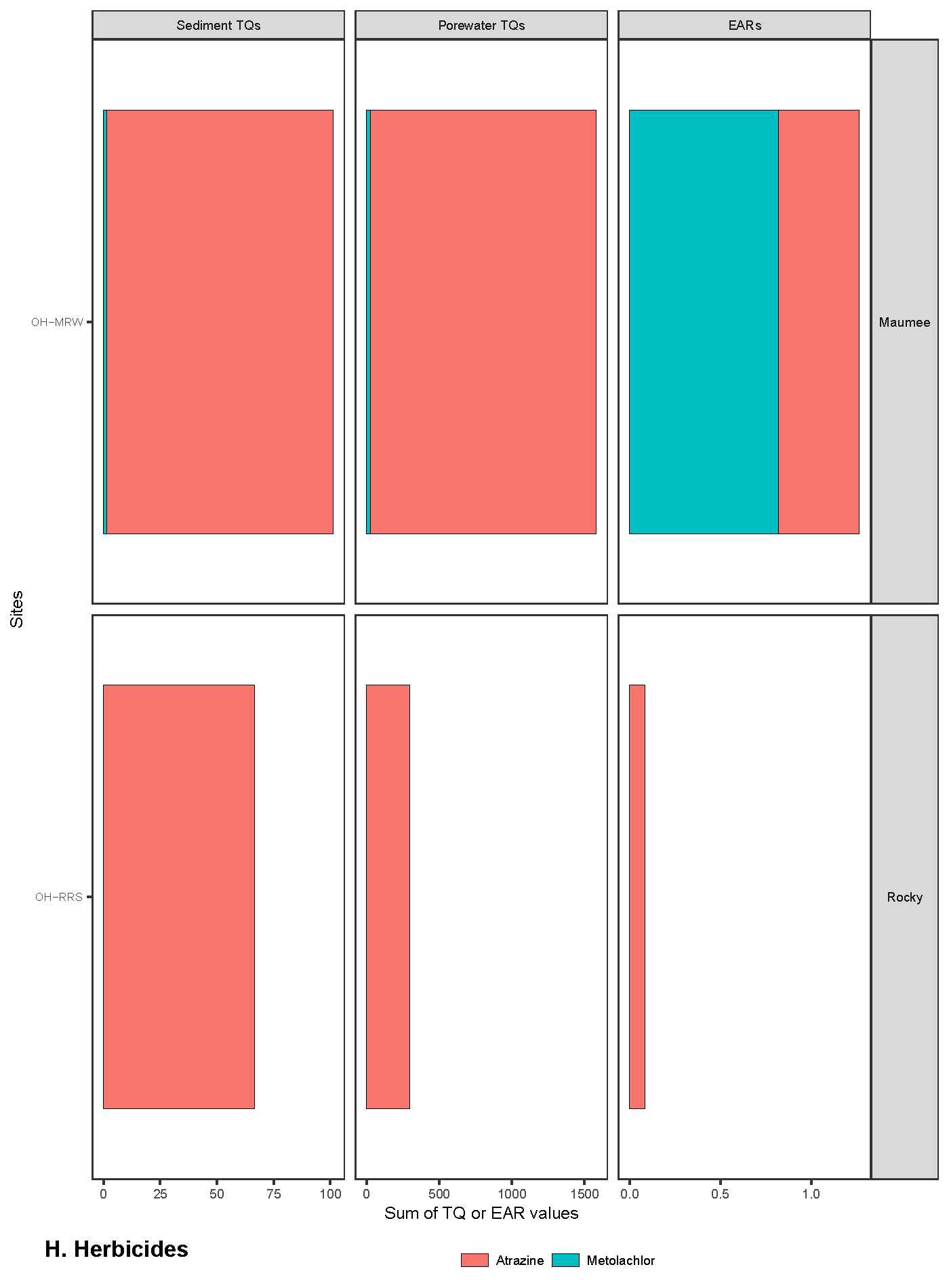


**SI Figure S5 H.** Summary of maximum (A) sediment and (B) porewater toxicity quotients (TQMax), and (C) exposure-activity ratios (EARMax) computed from organic waste compound concentrations in the herbicides class, measured in sediment samples from Great Lakes tributaries, 2017. Sites are grouped by watershed and within each watershed are listed upstream to downstream, top to bottom. Site abbreviations are defined in Table 1.


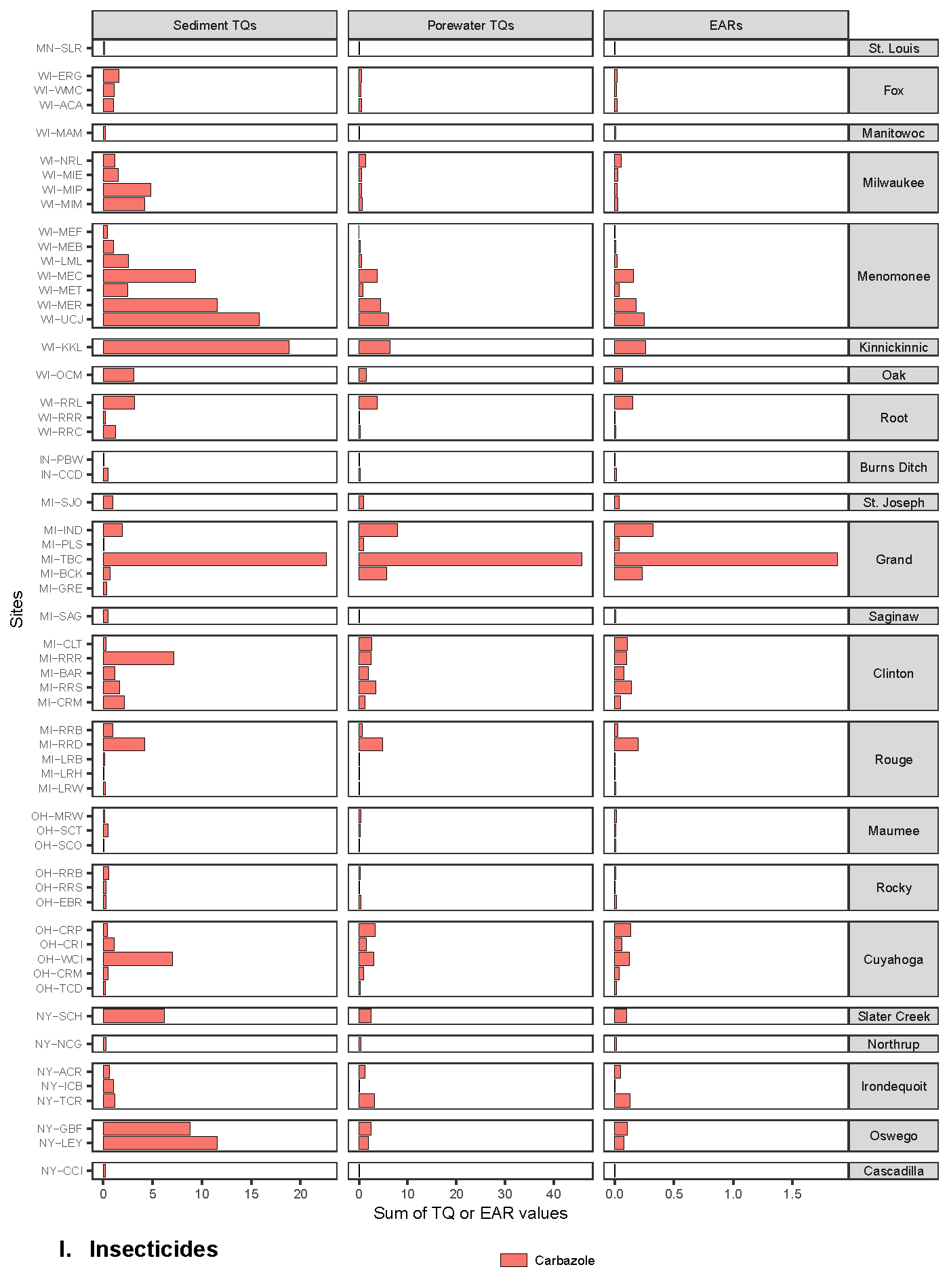


**SI Figure S5 I.** Summary of maximum (A) sediment and (B) porewater toxicity quotients (TQMax), and (C) exposure-activity ratios (EARMax) computed from organic waste compound concentrations in the insecticides class, measured in sediment samples from Great Lakes tributaries, 2017. Sites are grouped by watershed and within each watershed are listed upstream to downstream, top to bottom. Site abbreviations are defined in Table 1.


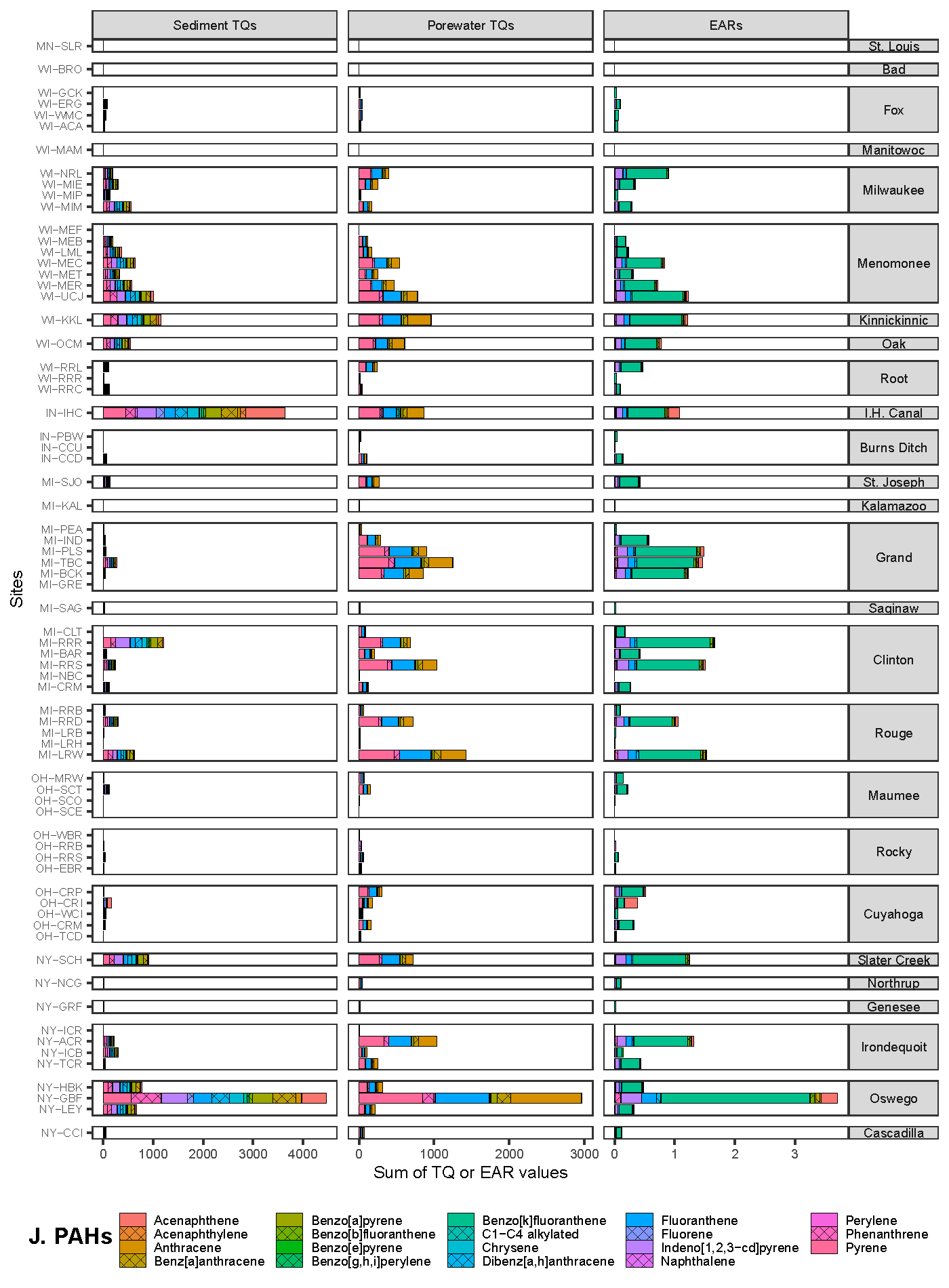


**SI Figure S5 J.** Summary of maximum (A) sediment and (B) porewater toxicity quotients (TQMax), and (C) exposure-activity ratios (EARMax) computed from organic waste compound concentrations in the polycyclic aromatic hydrocarbons (PAHs) class, measured in sediment samples from Great Lakes tributaries, 2017. Sites are grouped by watershed and within each watershed are listed upstream to downstream, top to bottom. Site abbreviations are defined in Table 1.


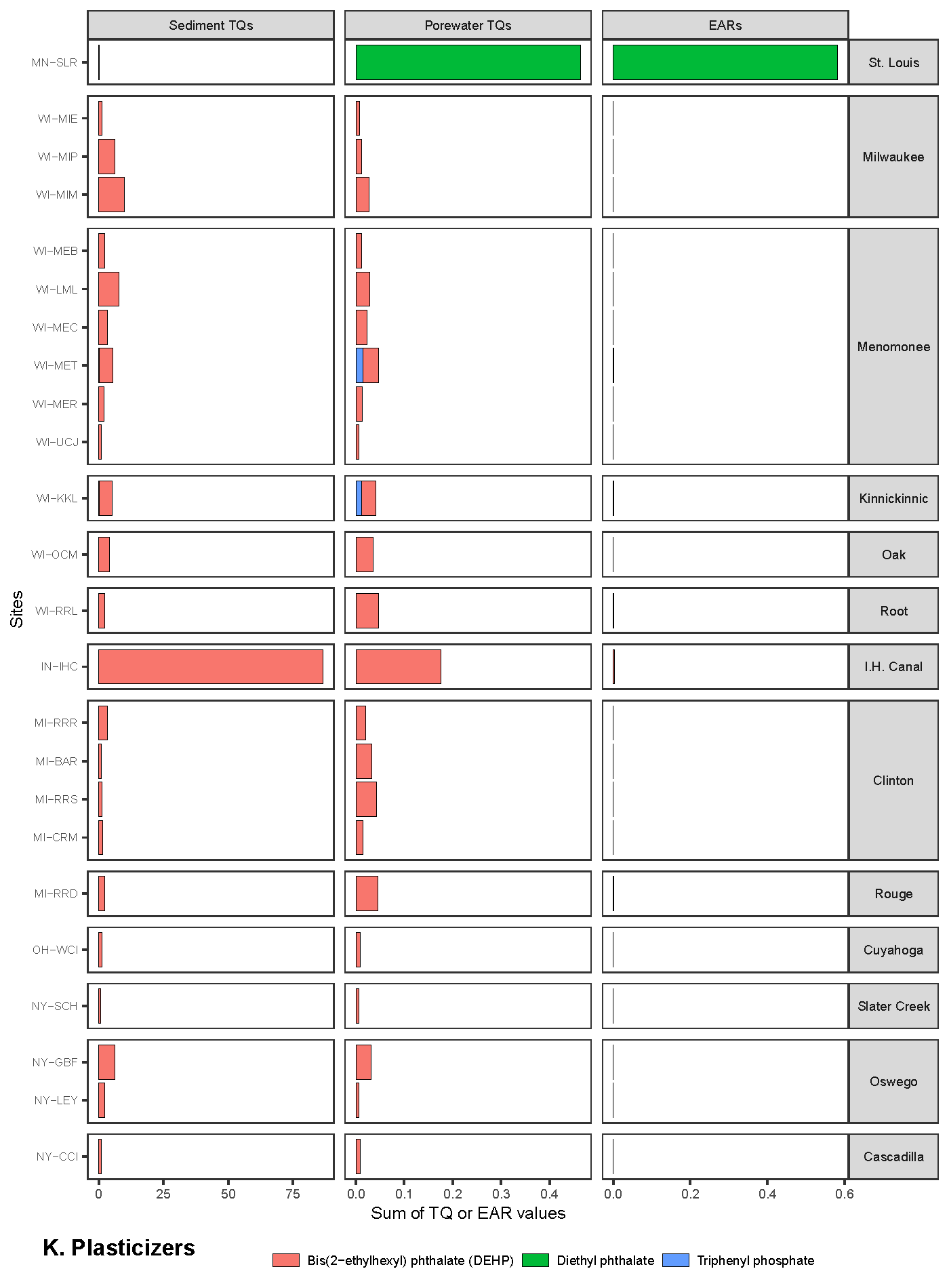


**SI Figure S5 K.** Summary of maximum (A) sediment and (B) porewater toxicity quotients (TQMax), and (C) exposure-activity ratios (EARMax) computed from organic waste compound concentrations in the plasticizers class, measured in sediment samples from Great Lakes tributaries, 2017. Sites are grouped by watershed and within each watershed are listed upstream to downstream, top to bottom. Site abbreviations are defined in Table 1.


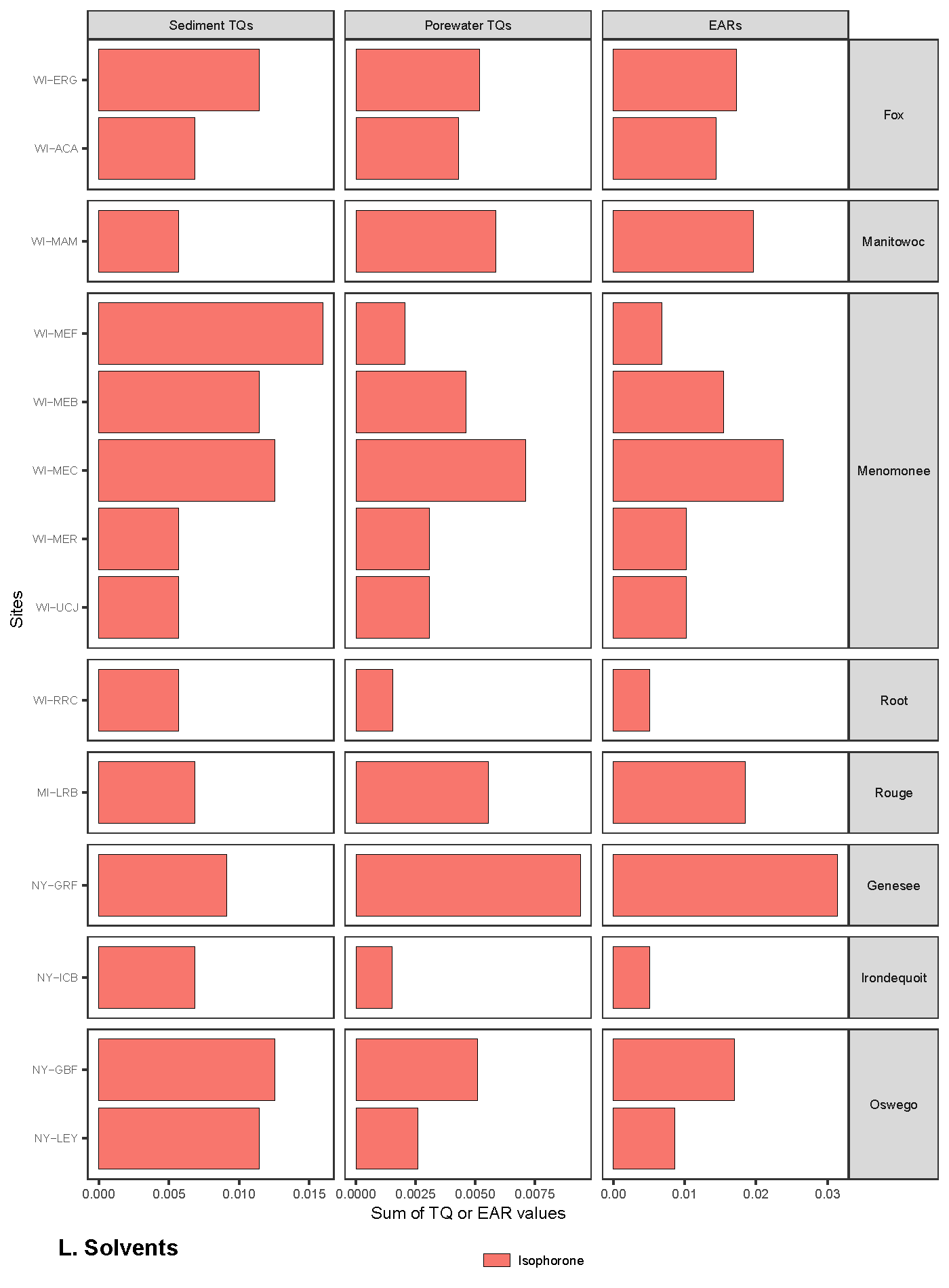


**SI Figure S5 L.** Summary of maximum (A) sediment and (B) porewater toxicity quotients (TQMax), and (C) exposure-activity ratios (EARMax) computed from organic waste compound concentrations in the solvents class, measured in sediment samples from Great Lakes tributaries, 2017. Sites are grouped by watershed and within each watershed are listed upstream to downstream, top to bottom. Site abbreviations are defined in Table 1.


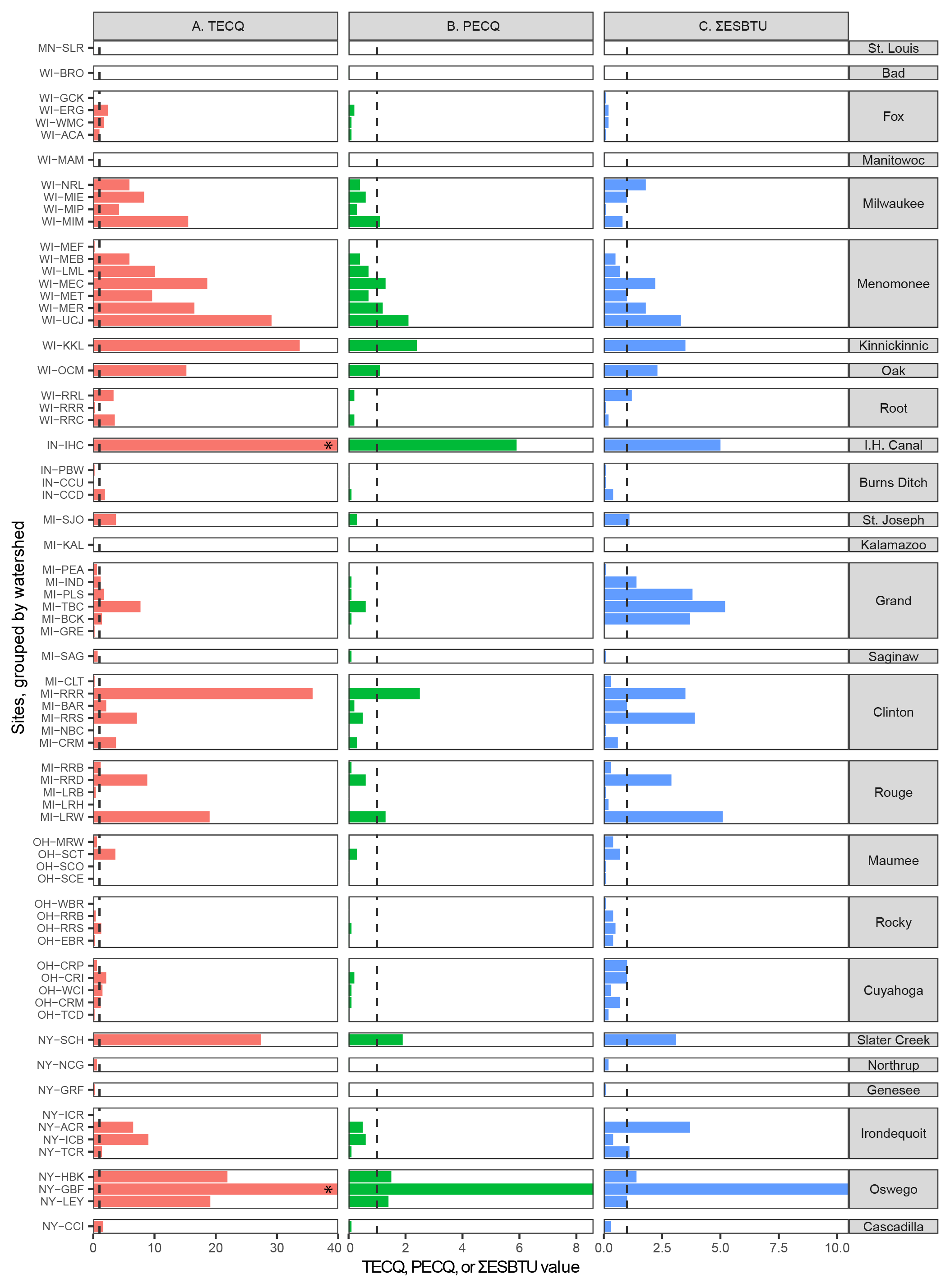


**SI Figure S6.** Potential toxicity of polycyclic aromatic hydrocarbon mixtures for stream sediment samples collected from Great Lakes tributaries, 2017 at each site using three assessment methods: (A) threshold effect concentration quotient (TECQ), (B) probable effect concentration quotient (PECQ), and (C) sum equilibrium partitioning sediment benchmark toxicity unit (ΣESBTU). TECQ and ΣESBTU values greater than 1.0 (dashed line) indicate the potential for adverse biological effects to sensitive benthic organisms, and PECQ values greater than 1.0 (dashed line) indicate probable adverse biological effects to sensitive benthic organisms. *TECQ bars for IN-IHC and NY-GBF were truncated to fit on plot, actual TECQ values are 83.6 and 122, respectively.


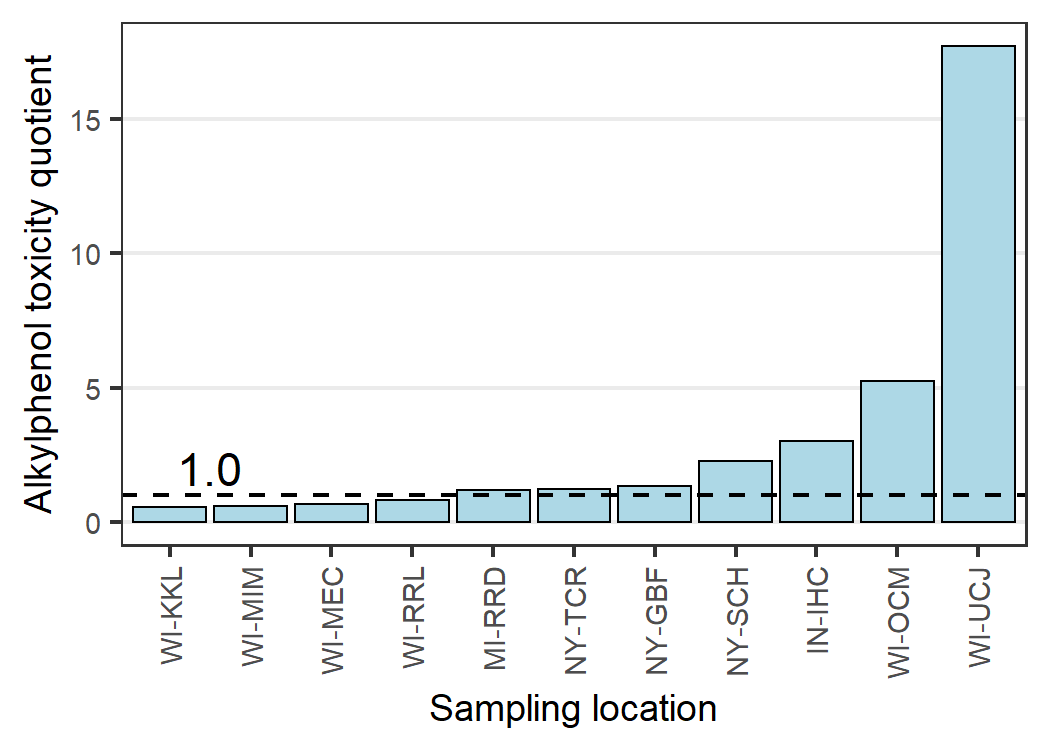


**SI Figure S7.** Potential toxicity of the mixture of alkylphenols at each site for stream sediment samples collected from Great Lakes tributaries, 2017. Toxicity is considered likely at values greater than 1.0.


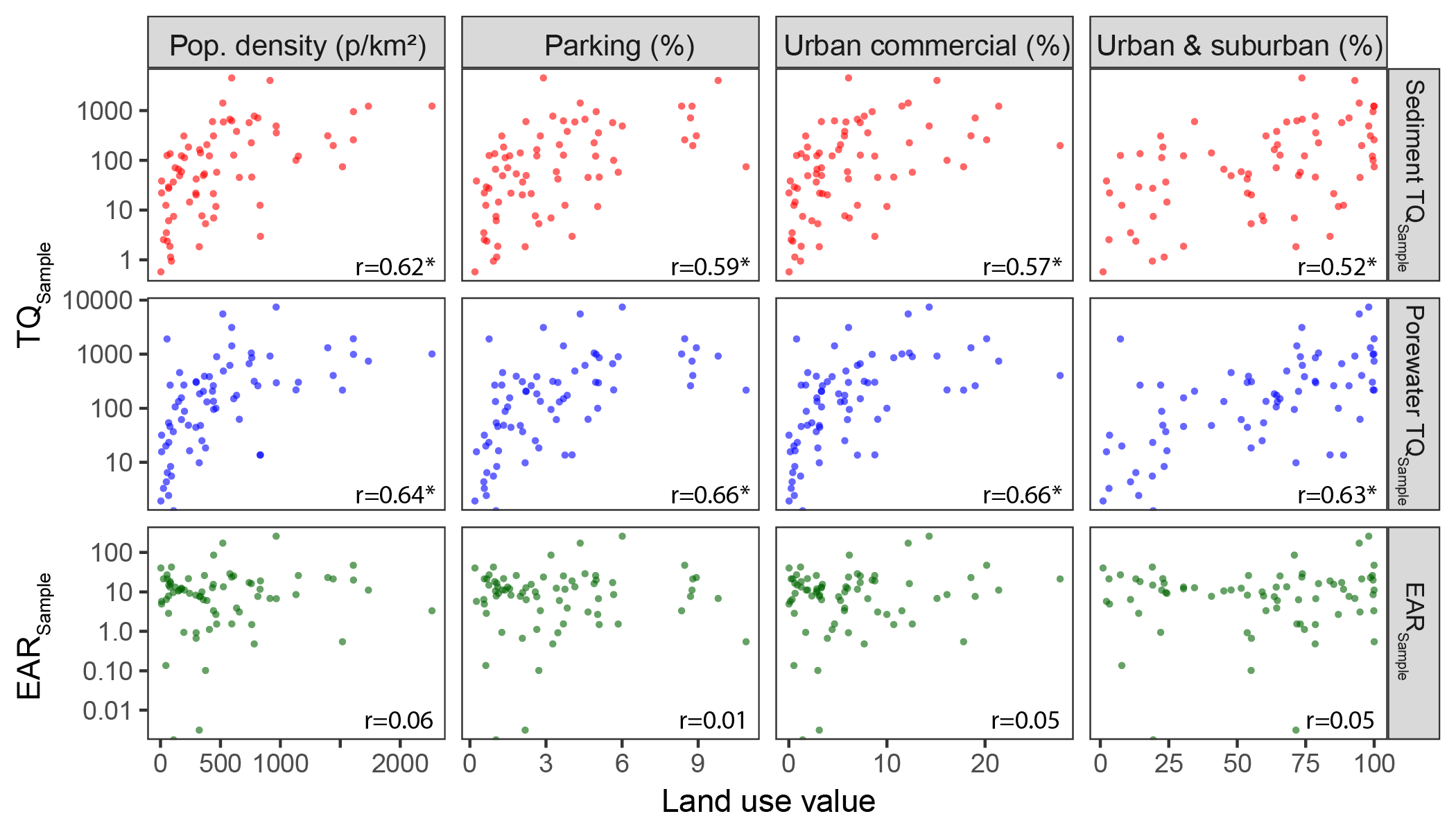


**SI Figure S8.** Relations between select watershed attributes and potential biological effects summed for each sample for stream sediment samples collected from Great Lakes tributaries, 2017. [TQ, toxicity quotient; TQ_Sample_, TQs summed for the chemicals in each sample; EAR, exposure-activity ratio; EAR_Sample_, EARs summed for the chemicals in each sample; p/km^2^, people per square kilometer; r, Spearman correlation coefficients; * p-values < 0.01]


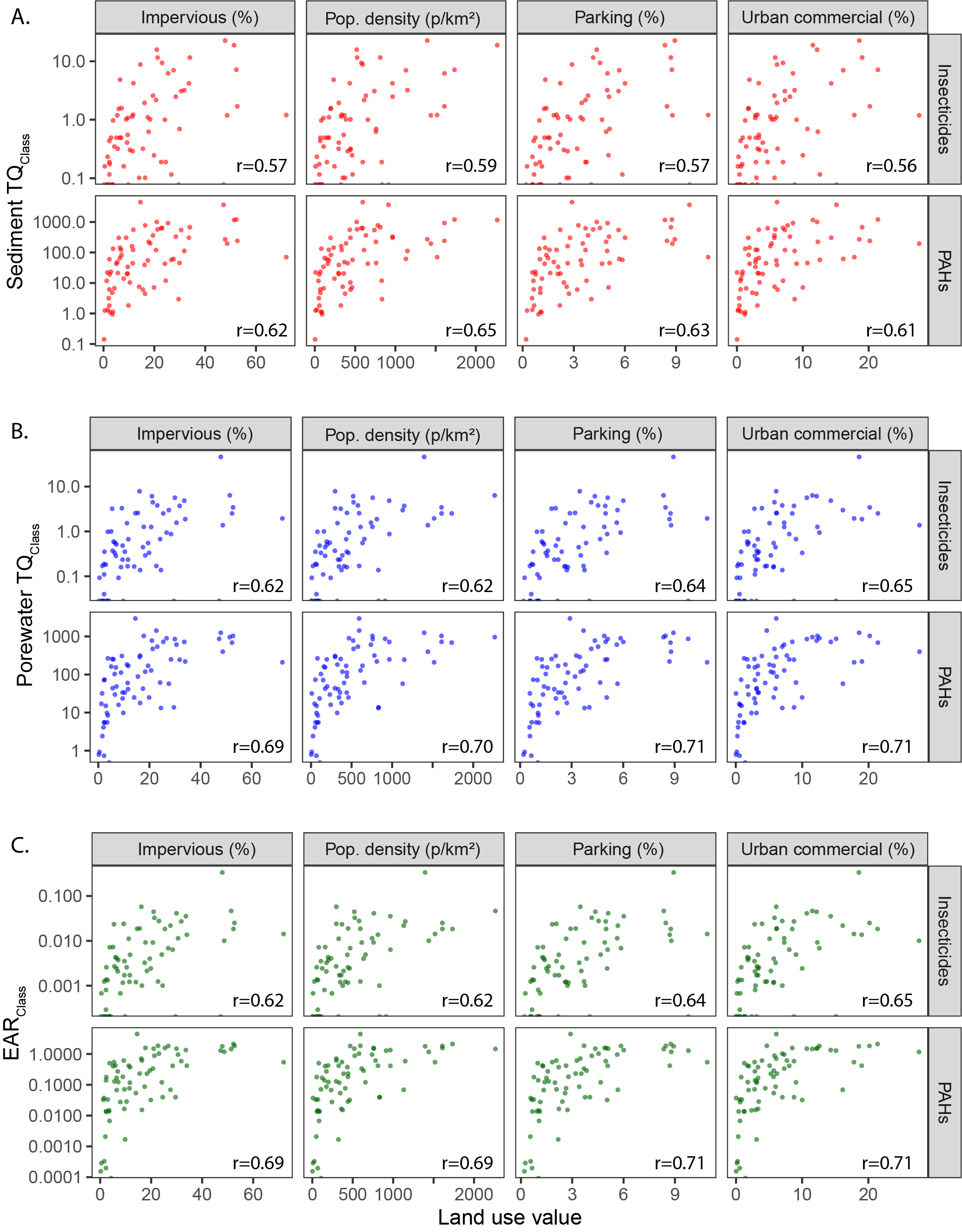


**SI Figure S9.** Relations between select watershed attributes and potential biological effects summed for select organic chemical classes for stream sediment samples collected from Great Lakes tributaries, 2017. Carbazole was the only insecticide detected. All Spearman correlation coefficients (r) have p-values < 0.01. [TQ_Class_, toxicity quotients summed for the chemicals in each class; EAR_Class_, exposure-activity ratios summed for the chemicals in each class; p/km^2^, people per square kilometer; PAHs, polycyclic aromatic hydrocarbons]


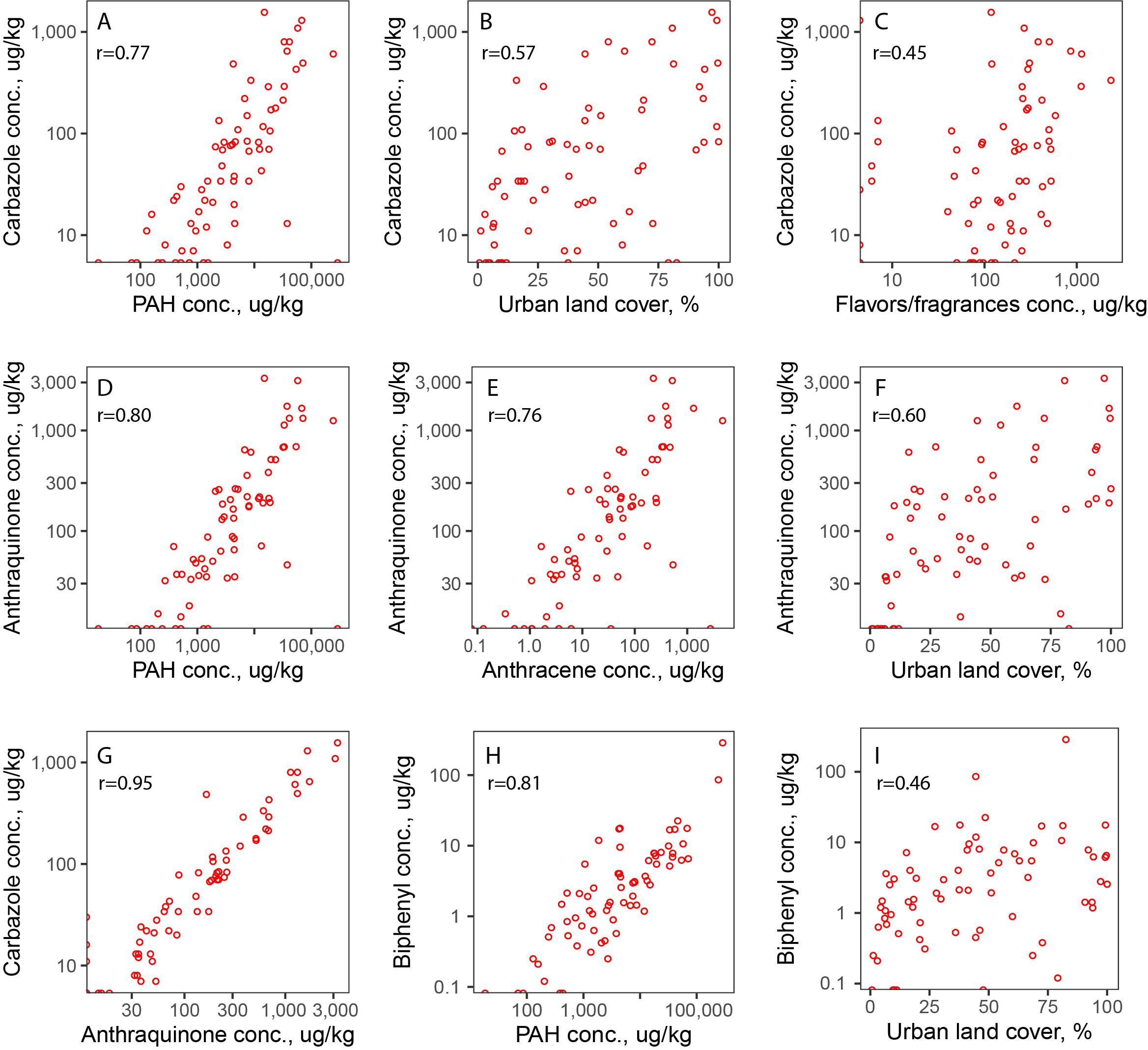


**SI Figure S10.** Relations between concentrations of individual chemicals (carbazole, anthraquinone, and biphenyl) and concentrations of other chemicals, summed concentrations of chemical classes, and watershed attributes for stream sediment samples collected from Great Lakes tributaries, 2017. Spearman correlation coefficients (r) have p-values < 0.01. [ug/kg, micrograms per kilogram, dry weight; PAH, polycyclic aromatic hydrocarbons; conc., concentration]

1. Any use of trade, firm, or product names is for descriptive purposes only and does not imply endorsement by the U.S. Government. [↑](#footnote-ref-1)
